# Contextual Integration in Cortical and Convolutional Neural Networks

**DOI:** 10.3389/fncom.2020.00031

**Published:** 2020-04-23

**Authors:** Ramakrishnan Iyer, Brian Hu, Stefan Mihalas

**Affiliations:** Modeling and Theory, Allen Institute for Brain Science, Seattle, WA, United States

**Keywords:** contextual modulation, convolutional neuronal network, canonical cortical microcircuit, inhibitory cell types, extraclassical receptive field, lateral connectivity, natural scene statistics, Bayesian inference

## Abstract

It has been suggested that neurons can represent sensory input using probability distributions and neural circuits can perform probabilistic inference. Lateral connections between neurons have been shown to have non-random connectivity and modulate responses to stimuli within the classical receptive field. Large-scale efforts mapping local cortical connectivity describe cell type specific connections from inhibitory neurons and like-to-like connectivity between excitatory neurons. To relate the observed connectivity to computations, we propose a neuronal network model that approximates Bayesian inference of the probability of different features being present at different image locations. We show that the lateral connections between excitatory neurons in a circuit implementing contextual integration in this should depend on correlations between unit activities, minus a global inhibitory drive. The model naturally suggests the need for two types of inhibitory gates (normalization, surround inhibition). First, using natural scene statistics and classical receptive fields corresponding to simple cells parameterized with data from mouse primary visual cortex, we show that the predicted connectivity qualitatively matches with that measured in mouse cortex: neurons with similar orientation tuning have stronger connectivity, and both excitatory and inhibitory connectivity have a modest spatial extent, comparable to that observed in mouse visual cortex. We incorporate lateral connections learned using this model into convolutional neural networks. Features are defined by supervised learning on the task, and the lateral connections provide an unsupervised learning of feature context in multiple layers. Since the lateral connections provide contextual information when the feedforward input is locally corrupted, we show that incorporating such lateral connections into convolutional neural networks makes them more robust to noise and leads to better performance on noisy versions of the MNIST dataset. Decomposing the predicted lateral connectivity matrices into low-rank and sparse components introduces additional cell types into these networks. We explore effects of cell-type specific perturbations on network computation. Our framework can potentially be applied to networks trained on other tasks, with the learned lateral connections aiding computations implemented by feedforward connections when the input is unreliable and demonstrate the potential usefulness of combining supervised and unsupervised learning techniques in real-world vision tasks.

## 1. Introduction

The visual response of a neuron [traditionally characterized by its classical receptive field (RF)] can be contextually modulated by visual stimuli outside the classical RF (Albright and Stoner, 2002). Such contextual effects are thought to be mediated in part by lateral connections between neurons in the same visual area/layer (providing near-surround modulation), as well as top-down feedback connections between neurons in different areas/layers (providing near and far-surround modulation) (Angelucci and Bressloff, 2006; Angelucci et al., 2017). Recent studies show non-random lateral connectivity patterns in the primary visual cortex (V1) of the mouse. Excitatory neurons with similar orientation tuning connect to each other with higher probability than to those tuned to the orthogonal orientation (Ko et al., 2011; Cossell et al., 2015; Lee et al., 2016). An even higher rate of connectivity is observed when their responses to natural scenes are well-correlated (Ko et al., 2011, 2013). This type of connectivity is consistent with a like-to-like, Hebbian wiring principle (Litwin-Kumar and Doiron, 2014; Sadeh et al., 2015a,b; Miconi et al., 2016; Zenke and Gerstner, 2017; Ocker and Doiron, 2018). In contrast, Bock et al. (2011) showed that inhibitory neurons receive non-specific, broadly tuned input from excitatory neurons. More recently, evidence for specific tuning of inhibitory neurons has also been presented (Znamenskiy et al., 2018). Connections from inhibitory neurons have been shown to be cell-type specific using both morphology-based (Jiang et al., 2015) and transgenic line-based cell-type identification (Pfeffer et al., 2013).

How does this observed lateral connectivity relate to proposed computations in cortical circuits? We present a normative network model in which every single pyramidal neuron implements Bayesian inference, combining evidence from its classical RF and from the near surround to estimate the probability of a feature being present^1^. We assume that the classical RF is formed by feedforward connections and the near surround effects of extra-classical RFs are mediated by lateral connections. We map feature probabilities to the steady-state firing rate of network neurons and show that the resultant lateral connections implementing this computation should depend on the covariances between unit activities. We limit ourselves to lateral connections between neurons with non-overlapping RFs in this study. Using natural image statistics (Martin et al., 2001) and electrophysiological data from mouse V1 (Durand et al., 2016), we show that the resulting lateral connectivity matrix qualitatively matches the experimentally reported like-to-like nature and distance dependence of connectivity in mouse visual cortex. We show that adding these lateral connections in an unsupervised manner to feedforward neural networks improves their performance on noisy image reconstruction and classification tasks. The computation naturally incorporates both divisive and subtractive inhibition. Inspired by the idea presented in Zhu and Rozell (2015) to model inhibitory interneurons in efficient sensory coding models using matrix decomposition techniques, we decompose the lateral connectivity matrices obtained with our model into low-rank and sparse components and relate these to different cell types. This enables us to explore the effects of cell-type specific perturbations on computations in artificial networks designed for reconstruction and classification tasks, suggesting a path to making them more biologically plausible (Marblestone et al., 2016).

## 2. Results

### 2.1. The Model

We assume a simple neural code for each excitatory neuron: the steady-state firing rate of the neuron maps monotonically to the probability of the feature that the neuron codes for being present in the image [similar to codes assumed in previous studies (Barlow, 1969; Anastasio et al., 2000; Rao, 2004)]. We have

(1) $$\begin{array}{l} f_{k,x}^{n}=g\left(p\left(F_{k}^{n}|i_{x}\right)\right) \end{array}$$

where $f_{k,x}^{n}$ represents the firing rate of a neuron coding for feature *F*_*k*_ at location *n* in image *x*, $p\left(F_{k}^{n}|i_{x}\right)$ represents the probability of presence of the corresponding feature and *g* is a monotonic function. For simplicity, we assume a linear mapping between the probability of feature presence and firing rate (*g*(*y*) = *y*) in the rest of the paper, as the qualitative conclusions are not dependent on this choice.

We note here that our model does not learn a dictionary of features, and works for arbitrary features, with a given set of constraints and approximations presented which we will mention throughout the construction of the model and summarize in the Discussion section. The application to more complex features is described with the incorporation of the model in convolutional neuronal networks, but to link to the biological structure we will start with simple features characteristic of early vision.

**Figure 1:**
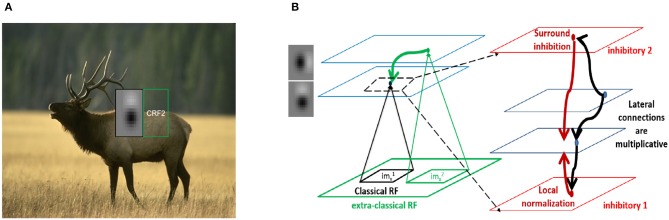
Contextual integration model. **(A)** Sample image from the BSDS dataset, with a superimposed classical receptive field (CRF1), and highlighting a surround patch (CRF2) with good predictive power about the feature in CRF1. **(B)** Classical receptive fields (small black and green squares) are the result of feedforward connections, while extra-classical receptive fields (larger green square) are assumed to be the result of lateral or feedback connections as shown on the left. The computation in Equation (4) requires a local circuit (expanded and shown on the right) with multiplicative lateral connections and two forms of inhibition: one for local normalization and one mediating the surround inhibition. This local circuit structure is assumed to be repeated at each spatial location (shown with blue squares).

An example of such a feature superimposed on a natural image is shown in Figure 1A. We subdivide the image into multiple patches corresponding to the size of the classical RF. We define the classical RF response of the neuron (with *g*(*y*) = *y*) as

(2) $$\begin{array}{l} c_{k,x}^{n}=p\left(F_{k}^{n}|i_{x}^{n}\right) \end{array}$$

where $i_{x}^{n}$ denotes the image patch at location *n*. We require that the sum of probabilities of all features in a patch is one, for every image, thereby implying a normalization of classical RF responses in a spatial region equal to the size of a patch so that ^2^

(3) $$\begin{array}{l} \underset{k}{\sum }c_{k,x}^{n}=1\;\forall n,x \end{array}$$

We show that a network of neurons can directly implement Bayes rule to integrate information from the surround (see Supplementary Information for the derivation). Intuitively, the activity of a neuron representing a feature is influenced by the probability that another feature is present in a surrounding patch and by the statistics of co-occurrence of these features. In such a network, the activity of a neuron representing feature *j* in patch *m*, given image *x*, can be shown to be (see Supplementary Information)

(4) $$\begin{array}{l} f_{j,x}^{m}=\frac{1}{N_{x}^{m}}c_{j,x}^{m}\overset{N}{\underset{n\neq m}{\prod }}\left(1+\underset{k}{\sum }W_{jk}^{mn}c_{k,x}^{n}\right) \end{array}$$

In Equation (4), $N_{x}^{m}$ represents a normalization coefficient (see Supplementary Information). The term $W_{jk}^{mn}$ represents a weight from the neuron coding for feature *k* in patch *n* to the neuron coding for feature *j* in patch *m* and can be estimated as:

(5) $$\begin{array}{l} W_{jk}^{mn}=\frac{\langle c_{j,x}^{m}c_{k,x}^{n}\rangle_{x}}{\langle c_{j,x}^{m}\rangle_{x}\langle c_{k,x}^{n}\rangle_{x}}-1 \end{array}$$

where *x* spans the set of images used and 〈.〉_*x*_ represents the average over all images in the set. Thus, lateral connections between neurons with non-overlapping RFs in our network are proportional to the relative probability of feature co-occurrences above chance in the set of images used.

While the formalism can be applied to any scene statistics, we focus here on the analysis of natural scenes. Equation (4) encapsulates a local computation of contextual integration by a network of excitatory neurons through *functional* lateral connections given by Equation (5).

### 2.2. Computation of Synaptic Weights

We generate a dictionary of simple cell like features by constructing a parameterized set of Gaussian filters from mouse V1 electrophysiological responses (Durand et al., 2016) (see Methods). We used natural images from the Berkeley Segmentation Dataset (Martin et al., 2001). To relate the activity of the neurons to the probability of a feature *F*_*k*_ being present in an image as in Equation (1), we convolve the image [after conversion to grayscale, normalizing to have a maximum value of 1 and subtraction of the average for each filter (Hyvärinen et al., 2001)] with the respective filters, rectify and normalize the convolved output in accordance with Equation (3) to get $c_{k,x}^{n}$ and estimate the connectivity using classical RF responses as in Equation (5). We assume translational invariance and limit the relative spatial position to three times the size of the classical RF (resulting in weights on a 43 × 43 grid) as the relative co-occurrence probabilities decrease significantly beyond this scale.

The resulting connectivity matrix *W*(*j, k*, Δ*x*, Δ*y*) is 4 dimensional, with the dimensions: cell type (*k*) of the source, cell type (*j*) of the target and relative spatial positions |*n* − *m*| ≡ (Δ*x*, Δ*y*) of the source and target cell types in the horizontal and vertical directions. Note that we are using the feature being coded for as a proxy for the excitatory cell type here. By construction, we have *W*(*j, k*, Δ*x*, Δ*y*) = *W*(*k, j*, Δ*x*, Δ*y*) so that the matrix is symmetric under exchange of source and target cell type.

**Figure 2:**
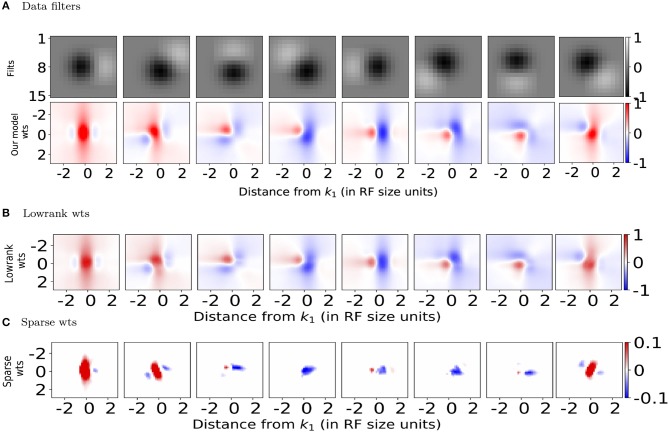
Spatial profiles of lateral connections. **(A)** Top: Subset of filters constructed (on 15 × 15 pixel grid) using estimates of spatial receptive field (RF) sizes from *in-vivo* recordings in mouse V1 (Durand et al., 2016), Bottom: Synaptic weights computed using Equation (5) onto the target neuron representing the left-most filter *k*_1_ in above row located at position ${\vec{x}}_{1}$ from the neurons representing filters *k*_2_ at position ${\vec{x}}_{2}$. **(B,C)** Lowrank and sparse components, respectively of weights shown in bottom panel of **(A)** (please see text for details). For all the weight heatmaps, axes represent distances from the center in terms of the RF size. Note that the colorbar for **(C)** ranges from −0.1 to 0.1 for clarity.

We present several 2D slices through the connectivity matrix (Figure 2A bottom row and Figure S3A second row). In addition to the dependence on differences in orientation tuning, the exact position and phase of the two neurons also contribute to the computation of the synaptic weights. In some cases, neurons with the same orientation tuning but different phases can have an inhibitory effect on each other (for example, panels 1 and 5 in both rows of Figure 2A). These results generalize well to other classical RF models, such as Gabor filters (which have been shown to be representative of RFs in cats and primates Jones and Palmer, 1987; Ringach et al., 2002; Ringach, 2004) as well as synthetic filters which are sharp/banded (see Figure S3).

### 2.3. Types of Inhibition and Relation to Mouse Cell Types

Two types of inhibition naturally arise in this computation (Figure 1B). The first is divisive normalization of excitatory neuronal activities (Equation 3), which could be implemented by the pyramidal (Pyr) targeting inter-neurons (PTI) category of Jiang et al. (2015) and corresponds well with the parvalbumin (PV)-expressing inter-neurons (Pfeffer et al., 2013). These neurons receive the average inputs of the pyramidal neurons whose RFs overlap with their classical RF and project back equally to them (see Supplementary Information).

The second type of inhibition arises in the computation of weights using Equation (5), which produces both positive and negative weights. These weights can be decomposed into excitatory and inhibitory components in various ways, with the simplest being a split into positive and negative parts. In an elegant study, Zhu and Rozell (2015) show that decomposing a recurrent excitatory connectivity matrix *G* (in a model of sparse coding) into a low-rank matrix (*L*) and a column-sparse matrix (*S*) [using an adaptive version of robust principal component analysis (Charles et al., 2013) (RPCA)] permits inhibitory interneurons having a diversity of tuning properties and characteristic E/I cell ratios. They suggest that *L* and *S* could be related to the PV and somatostatin (SOM) expressing mouse interneuron types, respectively. The technique exploits the fact that natural scene input statistics and models have low-dimensional structure. Motivated by this, we used a publicly available open-source library (Bouwmans et al., 2015; Sobral et al., 2015) and developed an adaptive version of the included RPCA algorithm based on the Principal Component Pursuit method (PCP) (Candès et al., 2011).

Following the convention in Zhu and Rozell (2015), the main idea involves solving the following convex optimization problem iteratively,

(6) $$\begin{array}{l} L,S={\text{arg min}}_{L,S}\|L{\|}_{*}+\|\Lambda S{\|}_{1}\text{subject to }G=L+S \end{array}$$

where ||.||_*_ is the sum of absolute values of eigenvalues (encouraging *L* to have lowrank) and ||.||_1_ is the *l*_1_ norm (sum of absolute values of the vectorized matrix) to encourage sparsity. Λ is a diagonal weighting matrix updated at each iteration using the rule $\Lambda_{ii}=\frac{\beta }{\|S^{\left(i\right)}{\|}_{1}+\gamma }$, where *S*^(*i*)^ is the *i*^*th*^ column of S, β controls competition between lowrank and sparsity and γ controls the speed of adaptation.

We used this to decompose the lateral connections (*W*) from our model into low-rank and sparse components (Figures 2B,C). Representing our connectivity matrix as *W*, we have *W* = *W*_*LR*_ + *W*_*S*_. The low-rank component can be decomposed using singular value decomposition as $W_{LR}=U\Sigma V^{T}$. *U*, *V* and *W*_*S*_ can be further separated, respectively into positive and negative components so that we have *W* = *W*_*LR*+_ + *W*_*LR*−_ + *W*_*S*+_ + *W*_*S*−_ with $W_{LR+}=U_{+}\Sigma V_{+}^{T}+\left(-U_{-}\right)\Sigma {\left(-V_{-}\right)}^{T}$ and $W_{LR-}=U_{-}\Sigma V_{+}^{T}+\left(-U_{+}\right)\Sigma {\left(V_{-}\right)}^{T}$.

We used γ = 1.0 for the learning rate and β = 0.01 to control the balance between lowrank and sparse. These were chosen such that the column-sparse matrix *W*_*S*_ was left with ~15% of non-zero entries compared to *W* and we retained only 14 of the 18 components in the SVD of *W*_*LR*_, retaining 99% of the variance in *W*_*LR*_. The different components in the decomposition can then be interpreted as disynaptic Pyr-Pyr connections (from *W*_*LR*+_), direct Pyr-Pyr connections (*W*_*S*+_), sparse (*W*_*S*−_) and lowrank (*W*_*LR*−_) disynaptic inhibition from surround Pyr neurons at relative spatial locations (Δ*x*, Δ*y*) onto center Pyr neurons.

In attempting to relate these different components and computations to cell types, we note that a large number of cell types have been characterized using transcriptomic methods by Tasic et al. (2018). In particular, they have observed a large diversity of SOM inhibitory subtypes. We propose that the low rank and sparse inhibitory components might correspond different SOM subtypes, with PV interneurons mediating divisive normalization as explained above.

### 2.4. Orientation and Distance Dependence of Connections

**Figure 3:**
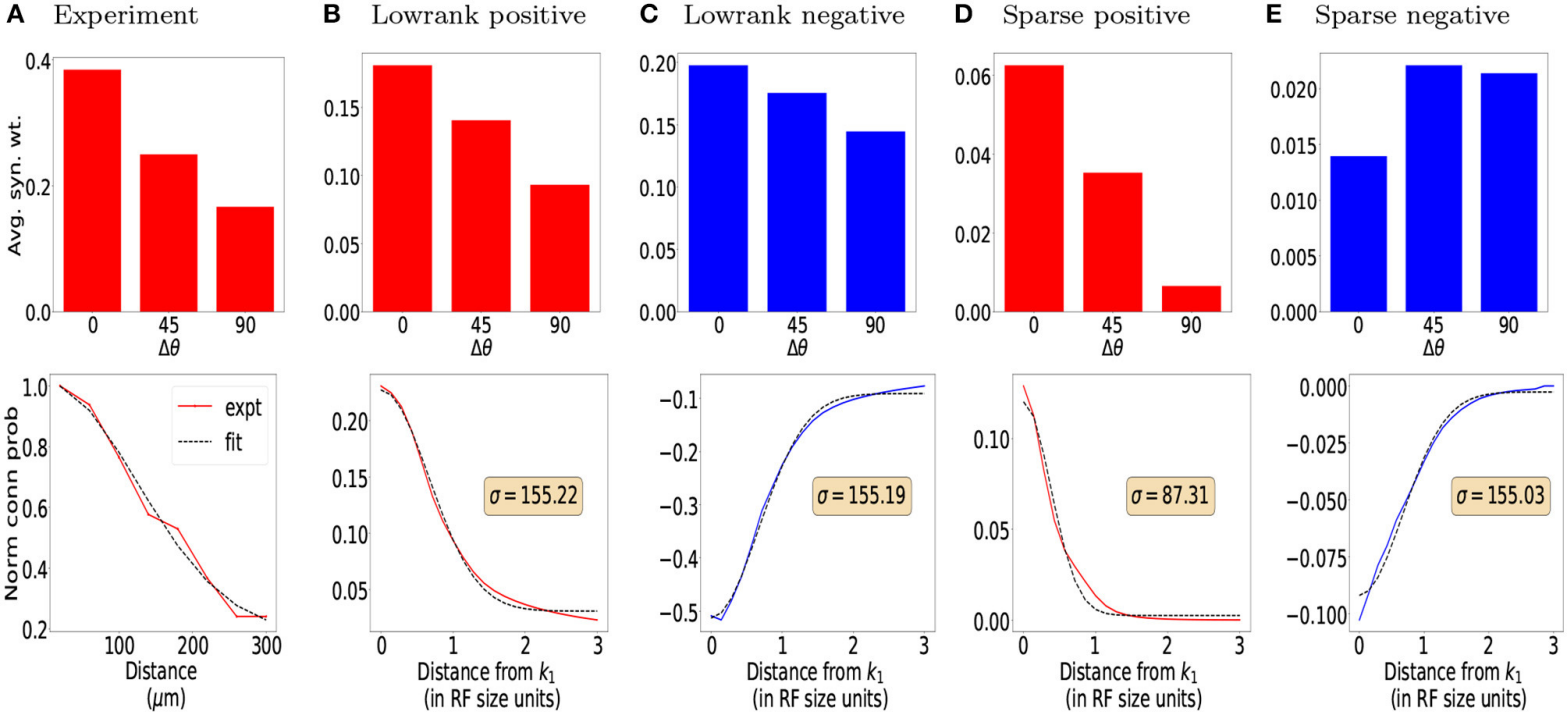
Orientation and distance dependence of synaptic weights for **(B,C)** Lowrank connections and **(D,E)** Sparse connections and comparison with experimental data. **(A)** Top: Connection probability as a function of difference in preferred orientation between excitatory neurons observed experimentally (from Ko et al., 2011). Bottom: Normalized connection probability between excitatory neurons as a function of inter-somatic distance as reported experimentally in mouse auditory cortex (Levy and Reyes, 2012) (see Methods for details). For **(B,D)** Top: Predicted average positive synaptic weights from our model as a function of difference in orientation. Bottom: Dependence of mean positive synaptic weights (connected points) on distance from RF center of target filter *k*_1_ and corresponding Gaussian fits for the positive weights (dashed black lines). For **(C,E)** Same as in **(B,D)**, for negative synaptic weights. In all plots, red bars/lines represent positive weights and blue bars/lines represent negative weights.

Both the lowrank and sparse excitatory connections (red bar plots in Figures 3B,D top row) obtained from our model show orientation dependence consistent with the connection probability (Figure 3A top panel) reported experimentally (Ko et al., 2011), with the sparse excitatory connections showing a stronger dependence on orientation tuning. The orientation dependence of lowrank and sparse inhibitory connection strengths is summarized in the top row (blue bars) of Figures 3C,E. The lowrank inhibitory connections' dependence is consistent with recent data on the weak orientation tuning of PV interneurons (Znamenskiy et al., 2018). Interestingly, our model predicts almost a non-specific dependence of the sparse inhibitory connections on the difference in orientation tuning, compared to the excitatory connections and could be tested experimentally.

The bottom rows of Figures 3B–E show the dependence of the mean positive (red lines) and negative (blue lines) synaptic weights, respectively onto a target neuron *k*_1_ from all neurons a fixed distance away, measured in terms of receptive field size. Using the cortical magnification of 30*deg*/*mm* (Garrett et al., 2014; Zhuang et al., 2017), the standard deviation of a Gaussian fit (Figures 3B,D, black dashed line, also see Methods) can be converted to σ_*lr*_ = 155 μ*m* and σ_*s*_ = 87 μ*m*, respectively, qualitatively similar to the measured distances (Levy and Reyes, 2012) of 114 μ*m* extrapolated from multi-patch recordings in mouse auditory cortex (Figure 3A bottom panel, see also Methods) and reported dependence in mouse visual cortex (Seeman et al., 2018). Both the lowrank and sparse inhibitory connections have a somewhat larger spatial extent than the excitatory connections (σ_*lr*_≈155μ*m*≈σ_*s*_), which could be verified experimentally. To the best of our knowledge, unlike in the rat somatosensory cortex (Silberberg and Markram, 2007; Berger et al., 2009), these disynaptic connections have not been measured directly in mouse cortex.

### 2.5. Application: Image Classification

The field of deep learning has traditionally focused on feedforward models of visual processing. These models have been used to describe neural responses in the ventral stream of humans and other primates (Cadieu et al., 2014; Güçlü and van Gerven, 2015; Yamins and DiCarlo, 2016; Wang and Cottrell, 2017) and have resulted in many practical successes (Gu et al., 2017). More recently, convolutional neural networks that include recurrent connections (both lateral and top-down) have also been proposed (Spoerer et al., 2017).

We incorporated lateral connections, learned in an unsupervised manner using our model, into multiple layers of convolutional neural networks which are trained in a supervised manner (network architectures used shown in Table S1). We first trained convolutional neuronal networks using standard backpropagation techniques. After training, we learned the lateral connections between units within a layer in an unsupervised manner. We show example learned lateral connections between different filters in the first convolutional layer (Figure S10).

**Figure 4:**
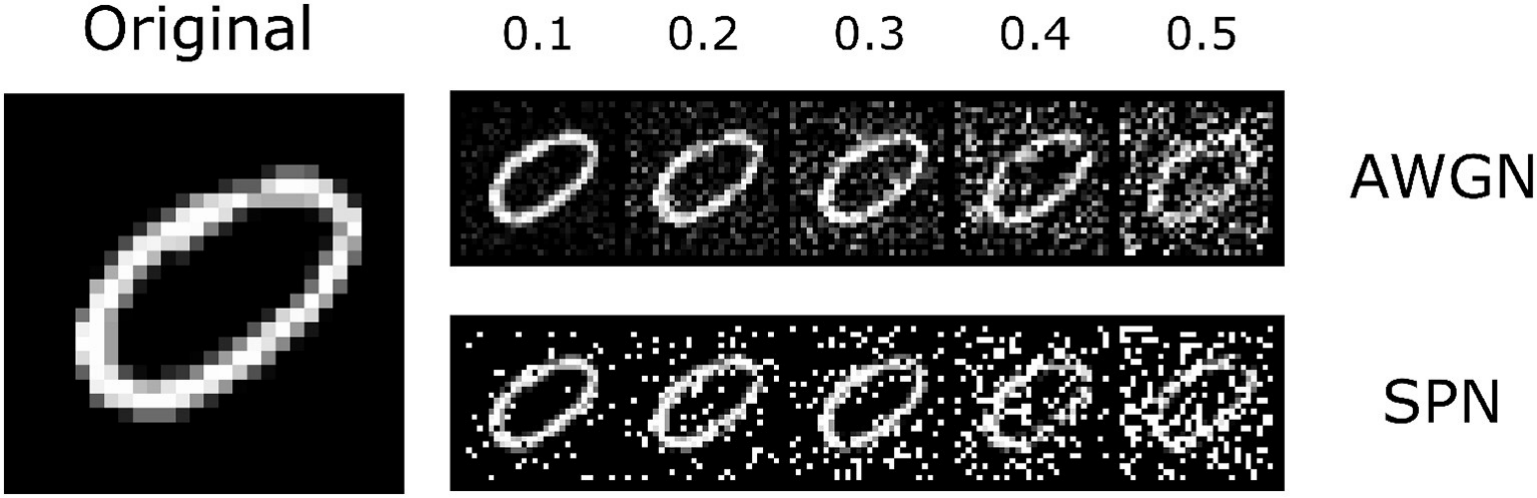
The MNIST dataset that was used in the experiments. Along with the original images, we introduced two types of noise perturbations: additive white gaussian noise (AWGN) and salt-and-pepper noise (SPN). An example image is shown to the left; the top row shows the AWGN stimuli, and the bottom row shows the SPN stimuli. Noise levels varied from 0.1 to 0.5 (increasing from left to right). The original image is reproduced from the MNIST (LeCun, 1998) dataset.

We tested our trained models with and without lateral connections on the original MNIST dataset (LeCun, 1998), as well as on noisy versions of this dataset (Figure 4). We hypothesized that lateral connections would provide the greatest benefit under noisy conditions, allowing units to integrate information from extra-classical receptive fields instead of relying solely on noisy feedforward input. To simplify computations, we assumed that contributions from the surround are sufficiently small and used a linearized form of Equation (4) for the firing rate,

(7) $$\begin{array}{l} f_{j,x}^{m,\left(l\right)}=c_{j,x}^{m,\left(l\right)}(1+\alpha \underset{k}{\sum }\underset{n\neq m}{\sum }W_{jk}^{mn,\left(l\right)}c_{k,x}^{n,\left(l\right)}) \end{array}$$

where the second term on the right side represents the contribution from the extra-classical RF, α represents a hyperparameter that tunes the strength of the lateral connections, and $W_{jk}^{mn,\left(l\right)}$ are the synaptic weights from surrounding units *n* on to unit *m* within layer *l*.

We find that both the base network and the network with lateral connections achieve high accuracy on the original test images (~98%). We also find that performance decreases gradually with increasing noise levels. In general, accuracy is lower for the salt-and-pepper noise (SPN) images compared to the additive white Gaussian noise (AWGN) images, suggesting that SPN images may be more difficult for the base model to handle. We find that lateral connections improve performance at higher levels of AWGN (standard deviations above 0.3) and also at higher levels of SPN (fraction of changed pixels above 0.1). We also tested decomposed versions of the lateral connections, by only using the low-rank or sparse components of the inhibitory weights. In general, the lateral connections seemed to improve performance of the model across different noise types, and furthermore, only using the sparse component of the inhibitory weights increased performance, suggesting a regularizing effect.

To check that model weights from Equation (5) indeed provide better functional results, for each layer, we replaced the weights with a uniform distribution of weights (*w* = 1/*N*_*T*_ where *N*_*T*_ is the total number of lateral connections in each layer). This leads to comparable results to the base model in the first row (CNN). Our results are summarized in Table 1. We provide an example of a separate application showing that lateral connections aid in image reconstruction in the Supplementary Information Section: Image Reconstruction.

**Table 1:** Model accuracy (%) on the MNIST dataset.

**Models**	**Original**	**AWGN**					**SPN**				
	**–**	**0.1**	**0.2**	**0.3**	**0.4**	**0.5**	**0.1**	**0.2**	**0.3**	**0.4**	**0.5**
CNN	**98.71**	**98.61**	**98.21**	**96.88**	92.03	81.78	97.28	92.01	80.85	65.29	48.28
CNNEx	97.25	97.17	96.83	95.86	93.34	88.24	96.06	93.45	87.97	77.99	63.04
CNNEx (avg)	98.71	98.58	98.15	96.83	91.89	81.90	**97.33**	92.11	80.79	64.87	47.94
CNNEx (lr)	97.25	97.18	96.83	95.87	93.37	88.29	96.08	93.49	87.99	78.00	63.10
CNNEx (s)	97.40	97.38	97.00	96.13	**93.80**	**88.84**	96.34	**93.93**	**88.44**	**78.46**	**63.47**

*We separate results for the original images and the two types of noise perturbations by columns (AWGN, additive white gaussian noise; SPN, salt-and-pepper noise). The results for the baseline model (CNN) and the model with lateral connections (CNNEx) are shown in the first two rows. The third row [CNNEx(avg)] shows results comparable to the baseline model (CNN) when we replaced the weights in Equation (5) with a uniform distribution of weights (w = 1/N_T_ where N_T_ is the total number of lateral connections in each layer). The last two rows, lr and s correspond to models with just the low-rank and just the sparse component, respectively of the inhibitory lateral connections. Including lateral connections seems to improve performance with increasing noise. Using only the sparse inhibitory component also increases performance, suggesting a regularizing effect. All reported values are averages over 10 random initializations*.

*Bold values represent highest accuracy for each case*.

Please note that when applying our formalism to such multi-layer networks (e.g., deep neural networks), we treat each feature map as containing units which respond to a given feature at a specific location within the image. For the first layer of the network (which sees the image as input), the learned lateral connections are captured by the derivations above. For deeper layers, we use the same formalism and set of assumptions, learning lateral connections between the hidden units based on their activations over a set of training images. During inference, we pass the real-valued activations modulated by the learned lateral connections onto the next layer (we do not perform any probabilistic sampling).

## 3. Discussion

We have presented a normative network model of cortical computation in which the lateral connections from surround neurons enable each center pyramidal neuron to integrate information from features in the surround. Our model predicts that the strength of lateral connections between excitatory neurons should be proportional to covariance of their activity in response to sensory inputs (Ko et al., 2014). Using the BSDS database of natural images and classical RFs parameterized using mouse V1 neuron responses, we find that excitatory neurons show like-to-like connectivity and distance dependence of connections in agreement with experiments.

We showed that adding these connections to deep convolutional networks in an unsupervised manner makes them more robust to noise in the input image and leads to better classification accuracy under noise. Including contributions from such lateral connections to noisy feedforward activity in a single-layer network also leads to better decoding performance. Intuitively, this suggests that under noisy conditions lateral connections enable each neuron to use available information from all surround neurons to provide the best possible representation it can.

The computation naturally suggests two forms of inhibition—local divisive normalization of excitatory neuronal activity in a patch (corresponding to classical RFs) and subtractive inhibition arising from the surround (extra-classical RFs). Decomposing the predicted lateral connectivity matrices for these networks into low-rank and sparse components allows us to relate the components to different cell types and explore the effects of cell-type specific perturbations on the performance of convolutional neural networks in an image classification task.

### 3.1. Relation to Previous Work

A number of normative and dynamical models relating contextual modulation of neuronal responses and lateral connectivity have been proposed in the literature. Normative models based on sparse coding (Olshausen and Field, 1996a,b, 1997; Bell and Sejnowski, 1997; Rozell et al., 2008; Zhu and Rozell, 2013, 2015) and its extension to spiking network models (Zylberberg et al., 2011; Shapero et al., 2014) predict anti-Hebbian lateral connections between excitatory neurons, in contrast with experimentally observed like-to-like excitatory connectivity. Such anti-Hebbian lateral connections can equivalently be implemented with a separate population of interneurons having Hebbian connectivity with excitatory neurons (King et al., 2013). The anti-Hebbian lateral connections arise as a consequence of feature competition induced by the sparsity constraint between dictionary elements with overlapping RFs at the same location.

Extensions of the sparse coding models have been proposed that give rise to like-to-like horizontal connections. Garrigues and Olshausen (2008) achieve this by including a pairwise coupling term in the prior for the sparse coding model. A recent study (Capparelli et al., 2019) achieves this by explicitly including spatial dependencies among dictionary elements with non-overlapping RFs into the sparse coding framework.

Other related normative models (Schwartz and Simoncelli, 2001; Karklin and Lewicki, 2009; Spratling, 2010; Coen-Cagli et al., 2012) propose different computational goals, while successfully capturing different aspects of observed lateral connectivity. Dynamical models with like-to-like recurrent connectivity (Li, 1998; Piëch et al., 2013) have also been developed to explain contour saliency (Li, 1999; Li and Gilbert, 2002) and to model perceptual organization in primates (Li, 2005; Mihalas et al., 2011). However, these models and their extensions do not include knowledge of the cell types involved and there is not an exact, formal description of the computations involved.

In contrast with these models, we are not building a statistical model of natural images and we are agnostic to the network-level computation which would determine the RFs. Instead, we are proposing that the local circuit—lateral connections between the excitatory neurons and their interactions with the inhibitory populations—provides contextual integration irrespective of the function implemented, which is encoded in the feedforward connections. This allows the circuit to be canonical, and have similar structure throughout cortex. The role of this local circuit is to allow the desired function to still be implemented with missing or partially corrupted inputs. While we limit our neuron functions to represent a feature from the previous feature map (which happens to be the input image for just the first layer in the network), this feature is in general arbitrary and we posit that each neuron performs inference for the presence of that feature, combining evidence from feed-forward (FF) connections with priors from lateral connections. We estimate weights from surround neurons (Equation 5) that would enable such inference. This allows us to incorporate our framework into any (multi-layer) network trained for specific tasks (e.g., digit classification in MNIST), with lateral connections (learned in an unsupervised manner) aiding the underlying computations when feedforward evidence is corrupted by input or neuronal noise. Given the appropriate classical RFs, we also expect our results to hold for different species (see Supplementary Information for results with Gabor RFs found in primates and cats) and cortical areas (in integrating information from different frequencies in auditory cortex, or locations in somatosensory cortex).

Similar to the above models, we show that our model is able to reproduce various aspects of physiology and contextual modulation phenomena. We provide comparisons with these other models where possible in the Supplementary Information.

### 3.2. Model Assumptions and Limitations

In sketching a proof for how a network of neurons can directly implement Bayes' rule to integrate contextual information, we have made some simplifying assumptions that limit the scope of applicability of our model. We discuss some of those here.

For simplicity, we have assumed a linear relationship between probability of feature presence and neuronal responses. While we use a simple filter model (ReLU + normalization) to model responses and connectivity in mouse V1, our basic theoretical argument holds for any set of features on the previous feature map. In the CNNs, the same principle is applied at multiple layers in depth where the representations are highly non-linear. We chose a relatively simple dataset and network architecture as a proof-of-concept for our model. Future experiments will have to test the scalability of learning optimal lateral connections on more complex network architectures and larger image datasets [e.g., ImageNet (Deng et al., 2009)], and whether these connections provide any benefit against noise or other types of perturbations, such as adversarial images.

Many probabilistic models of cortical processing have multiple features at each location that contribute to generating an image patch, but not all of them require probabilities to sum to one (for eg, sparse coding) unlike our model. In contrast, our model is not a generative model for natural image patches. Interactions between neurons at the same location arise (via divisive normalization) in our model as a consequence of requiring probabilities to sum to one, leading to feature competition. We note that integration of sparse coding models with our model is possible, but beyond the scope of this study.

For each location, we only derive the connections from surrounding neurons onto the center neuron, without higher-order effects of the reverse connections from the center to the surround neurons. The proof to derive Equation (4) also requires the inputs to the neurons to be independent. One simple way to achieve such independence is to have non-overlapping classical receptive fields. Practically, we have observed that relaxing the requirement of independence, as it was done for the CNN analysis which include connections between neurons with partly overlapping RFs, continues to result in significant improvement in the function of the network.

To simplify computations involved with testing the performance of CNNs with lateral connections included, we linearized the expression in Equation 4 by assuming that contributions of lateral connections from each patch are not very large. As a quick estimate, we computed the effect of lateral interactions for every point in 200 natural images, and find they have a mean of 0.03 and a standard deviation of 0.12.

Typically, models with lateral interactions amount to a recurrent network eliciting waves of activation (Muller et al., 2018). As our lateral connections are balanced, with each connection having the same delay, and are relatively small, running once though the recurrent loop allows for fast processing without deviating too far from the recurrent network. We thus believe that we are justified in using a feed-forward model to include lateral interactions.

Even accounting for these assumptions and limitations, our simple model provides good qualitative and quantitative agreement with experimental observations in mouse cortex and provides experimentally testable predictions for connectivity between different cell types. Incorporating such biologically inspired lateral connections in artificial neural networks also aids in their performance, especially in the presence of noisy inputs. Our framework demonstrates how supervised and unsupervised learning techniques can be combined in vision-based artificial neural networks and can be easily adapted to networks trained on other tasks.

## 4. Methods

### 4.1. Classical Receptive Field Parameterization

Filters were constructed on a 15 × 15 spatial grid. We summed up the area under all pixels whose intensities were >95% of the maximum pixel to get an effective area *A* and effective radius *r* using *A* = π*r*^2^ for each filter in the basis set. The filter size was computed as the mean radius of all basis filters. Basis filters were constructed by averaging estimates of spatial receptive field (RF) sizes from 212 recorded V1 cells (Durand et al., 2016). They consisted of four types of spatial RFs observed experimentally: ON only, OFF only and two versions of ON/OFF cells with the first having a stronger ON subfield and the second a stronger OFF subfield. Using the average sizes of all recorded V1 units, we modeled each subfield as a 2D Gaussian with standard deviation σ = 0.5 × average subfield size, which was measured to be 4.8° for the OFF subfields and 4.2° for the ON subfields. The relative orientation between the two subfields for each ON/OFF class was varied from 0 to 315° uniformly in steps of 45°, resulting in a total of 18 basis filters. For the ON/OFF class, the distance between the centers of the two subfields was chosen to be 5° (which equates to roughly 2σ). In accordance with data, the amplitude of the weaker subfield was chosen to be half that of the stronger subfield (which was set to be 1). The two subfields were then combined additively to form a receptive field. The size of these filters was estimated to be *r*≈7°.

### 4.2. Distance Dependence of Synaptic Weights

To draw the plot in Figure 3A (bottom panel) for the experimentally measured data from mouse auditory cortex (Levy and Reyes, 2012), we used an open-source freely available graphics software called GraphClick http://www.arizona-software.ch/graphclick/. We obtained the normalized connection probability as a function of the reported distances in Levy and Reyes (2012). We quantified the distance dependence of mean positive and negative synaptic weights obtained from our model as follows. We first computed ${\bar{W}}_{\pm }\left(\Delta x,\Delta y\right)={\langle W_{\pm }\left(\Delta x,\Delta y,k_{1},k_{2}\right)\rangle }_{k_{1},k_{2}}$ and then computed the average of ${\bar{W}}_{\pm }\left(r\left(\Delta x,\Delta y\right)\right)$ for all points on the square given by *r* = *max*(|(Δ*x*)|, |(Δ*y*)|) on the (43 × 43) grid of synaptic weights. We fit a Gaussian function of the form $\bar{w}=w_{m}\;\exp\;\left(-\frac{r^{2}}{2\sigma^{2}}\right)+w_{0}$. Here, $\bar{w}$ represents the normalized mean synaptic weight from our model as a function of the distance *r*. The parameters (*w*_*m*_, *w*_0_, σ) are respectively the amplitude, dc offset and standard deviation of the Gaussian. We optimized for these three parameters using the SciPy curve_fit function in Python.

### 4.3. Adding Lateral Connections to Deep Convolutional Networks

We trained and evaluated our models on the MNIST (LeCun, 1998) dataset. To test the generalization of our models under noise, we added two types of noise to the original images: additive white Gaussian noise and salt-and-pepper noise. We used a network architecture consisting of two convolutional layers, each followed by a max-pooling operation, and two fully-connected layers, with the final output passed through a soft-max non-linearity. The ReLU non-linearity was used after all other layers. Ten models with different random seeds were trained using stochastic gradient descent. To learn the lateral connections, we applied (5) to the activations of the first two convolutional layers over the set of training images, while keeping the weights of all filters fixed. During inference (Equation 7), the contribution from lateral connections are scaled by an additional α parameter, whose value was chosen based on a held-out set of validation data. Decomposition into lowrank and sparse components for lateral connections used β = 0.1 and β = 0.25 for the two convolutional layers. All reported results are averages over the 10 trained models.

## Data Availability Statement

The datasets generated for this study are available on request to the corresponding author.

## Author Contributions

SM designed and supervised the study and developed the theoretical framework. RI implemented the theory, the matrix decomposition and relation to cell types, carried out comparisons with experiments, phenomenology, and previous studies, and contributed to simulations with the multi-layer neural network. BH implemented the multi-layer neural network and applied the theory to image classification. RI, BH, and SM wrote the manuscript.

## Conflict of Interest

The authors declare that the research was conducted in the absence of any commercial or financial relationships that could be construed as a potential conflict of interest.

## Footnotes

^1^Several proposals for how neurons might represent probabilities have been presented (Pouget et al., 2013), the simplest of which directly relate neural activity to the probability of a feature being present in the neuron's classical RF (Barlow, 1969; Anastasio et al., 2000; Rao, 2004).

^2^In practice, we add a small constant ϵ to the sum on the left before normalizing. This is equivalent to a null feature for when no substantial contrast is present in patch *n*.
