# Erratum: Contextual Integration in Cortical and Convolutional Neural Networks

**DOI:** 10.3389/fncom.2020.00067

**Published:** 2020-07-07

**Authors:** Frontiers Production Office

**Affiliations:** Frontiers Media SA, Lausanne, Switzerland

**Keywords:** contextual modulation, convolutional neuronal network, canonical cortical microcircuit, inhibitory cell types, extraclassical receptive field, lateral connectivity, natural scene statistics, Bayesian inference

Due to a production error, there was a mistake in Table 1 as published. The bold entries indicating the highest accuracy for each case were un-bolded erroneously except for column SNP, 0.1. The correct Table 1 with bold values in each column appears below.

**Table 1:** Model accuracy (%) on the MNIST dataset.

**Models**	**Original**	**AWGN**					**SPN**				
	**–**	**0.1**	**0.2**	**0.3**	**0.4**	**0.5**	**0.1**	**0.2**	**0.3**	**0.4**	**0.5**
CNN	**98.71**	**98.61**	**98.21**	**96.88**	92.03	81.78	97.28	92.01	80.85	65.29	48.28
CNNEx	97.25	97.17	96.83	95.86	93.34	88.24	96.06	93.45	87.97	77.99	63.04
CNNEx (avg)	98.71	98.58	98.15	96.83	91.89	81.90	**97.33**	92.11	80.79	64.87	47.94
CNNEx (lr)	97.25	97.18	96.83	95.87	93.37	88.29	96.08	93.49	87.99	78.00	63.10
CNNEx (s)	97.40	97.38	97.00	96.13	**93.80**	**88.84**	96.34	**93.93**	**88.44**	**78.46**	**63.47**

*We separate results for the original images and the two types of noise perturbations by columns (AWGN, additive white gaussian noise; SPN, salt-and-pepper noise). The results for the baseline model (CNN) and the model with lateral connections (CNNEx) are shown in the first two rows. The third row [CNNEx(avg)] shows results comparable to the baseline model (CNN) when we replaced the weights in Equation (5) with a uniform distribution of weights (w = 1/N_T_ where N_T_ is the total number of lateral connections in each layer). The last two rows, lr and s correspond to models with just the low-rank and just the sparse component, respectively of the inhibitory lateral connections. Including lateral connections seems to improve performance with increasing noise. Using only the sparse inhibitory component also increases performance, suggesting a regularizing effect. All reported values are averages over 10 random initializations*.

*Bold values represent highest accuracy for each case*.

The publisher apologizes for this mistake. The original article has been updated.

