# Supplementary material for: Contextual Integration in Cortical and Convolutional Neural Networks
Source: Front Comput Neurosci. 2020 Apr 23;14:31. doi: 10.3389/fncom.2020.00031 (PMC7192314; doi:10.3389/fncom.2020.00031)
Supplement: Supplementary file 1 [file Data_Sheet_1.PDF]

Contextual integration in cortical and convolutional neural networks

Ramakrishnan Iyer, Brian Hu, Stefan Mihalas

March 17, 2020

1 Supplementary Information

All SI figures are provided at the end of SI text, with each figure on a new page.

Proof of contextual integration

To keep the proof of context integration self-contained, we reproduce some of the material already introduced at the beginning of the Results section here.

Neuronal code

We assume a simple neural code for each excitatory neuron: the steady-state firing rate of the neuron maps monotonically to the probability of the feature that the neuron codes for in the presented image (similar to codes assumed in previous studies [1–3]). We have

$$f^n_{k,x} = g(p(F^n_k|i_x)) \tag{S1}$$

where $f^n_{k,x}$ represents the firing rate of a neuron coding for feature F_k at location n in response to image x, $p(F^n_k|i_x)$ represents the probability of feature F_k being present at location n in image x and g is a monotonic function. For simplicity, we assume a linear mapping between the probability of feature presence and firing rate ($g(y) = y$), as the qualitative conclusions are not dependent on this choice.

We subdivide the image into multiple sections corresponding to the sizes of classical receptive fields and refer to each section as a patch. We define the classical receptive field response of the neuron (with $g(y) = y$) as

$$c^n_{k,x} = p(F^n_k|i^n_x) \tag{S2}$$

where i^n_x denotes the image patch at location n. We require that the sum of probabilities of all features in a patch is one, for every image, thereby implying a normalization of classical receptive field responses in a spatial region.

$$\sum_k c^n_{k,x} = 1 \quad \forall n, x \tag{S3}$$

Proof

We subdivide the image into N patches corresponding to the sizes of classical receptive fields.

$$p(F^n_k|i_x) = p(F^n_k|i^1_x, ..., i^N_x) \tag{S4}$$

For clarity, we first look at the integration of information from two patches, where patch i^1_x is in the classical RF for the neuron considered and patch i^2_x is in its extra classical RF. We start by considering how the code for feature j at location 1 (F^1_j) within patch i^1_x is influenced by the patch i^2_x.

For these two patches, assuming that the set of features represents a complete basis, (**S4**) can be expanded as:

$$p(F_j^1|i_x^1, i_x^2) = \sum_k p(F_j^1|i_x^1, i_x^2, F_k^2)\ p(F_k^2|i_x^1, i_x^2) \quad \textbf{(S5)}$$

where the sum is over the k neurons with classical receptive fields in patch i_x^2. We make the following simplifying assumptions:

1. The only information that neuron 1 has about patch i_x^2 comes from the k neurons with classical receptive fields in that patch, so that,

   $$p(F_j^1|i_x^1, i_x^2, F_k^2) \approx p(F_j^1|i_x^1, F_k^2). \quad \textbf{(S6)}$$

   Using Bayes rule, we can write

   $$p(F_j^1|i_x^1, F_k^2) = \frac{p(F_j^1|i_x^1)\ p(F_k^2|i_x^1, F_j^1)}{p(F_k^2|i_x^1)}. \quad \textbf{(S7)}$$

2. Note that the term $p(F_k^2|i_x^1)$ represents the extraclassical RF influence of patch i_x^1 onto the neuron coding for feature F_k^2. The term $p(F_k^2|i_x^1, F_j^1)$ represents the joint influence of patch i_x^1 and the activity of neuron of interest (in this case, the neuron coding for featureF_j^1) onto the neuron coding for feature F_k^2. In this study we will consider only the first order dependence and exclude higher-order dependencies by making the following set of simplifying assumptions for these terms:

   $$\begin{aligned} p(F_k^2|i_x^1, F_j^1) &\approx p(F_k^2|F_j^1) \quad \textbf{(S8)} \\ p(F_k^2|i_x^1) &\approx p(F_k^2) \\ p(F_k^2|i_x^1, i_x^2) &\approx p(F_k^2|i_x^2) \end{aligned}$$

   Using these assumptions, we can rewrite (**S7**) as,

   $$p(F_j^1|i_x^1, F_k^2) = \frac{p(F_j^1|i_x^1)\ p(F_k^2 \cap F_j^1)}{p(F_j^1)\ p(F_k^2)} \quad \textbf{(S9)}$$

   Substituting (**S6**) in (**S5**) and using (**S9**), we can write,

   $$\begin{aligned} p(F_j^1|i_x^1, i_x^2) &= \sum_k p(F_j^1|i_x^1, F_k^2)\ p(F_k^2|i_x^1, i_x^2) \\ &= p(F_j^1|i_x^1) \sum_k \left(1 + \frac{p(F_j^1 \cap F_k^2) - p(F_j^1)\ p(F_k^2)}{p(F_j^1)\ p(F_k^2)}\right) p(F_k^2|i_x^2) \quad \textbf{(S10)} \end{aligned}$$

   Given the normalization condition in (**S3**), the re-arrangement of terms in (**S10**) allows us to split the contributions as a sum of classical (feed-forward) and extra-classical (lateral) terms so that

   $$p(F_j^1|i_x^1, i_x^2) = p(F_j^1|i_x^1) \left(1 + \sum_k \frac{p(F_j^1 \cap F_k^2) - p(F_j^1)\ p(F_k^2)}{p(F_j^1)\ p(F_k^2)} p(F_k^2|i_x^2)\right) \quad \textbf{(S11)}$$

3. When going beyond two patches to N patches, we use a third approximation: the patches considered do not overlap and each of these patches provides independent information to the neuron coding for feature F_j in patch 1. The probability of feature F_j in patch 1 becomes:

   $$\begin{aligned} p(F_j^1|i_x) &= p(F_j^1|i_x^1, ..., i_x^N) \\ &= p(F_j^1|i_x^1) \prod_{n \neq 1}^{N} \left(1 + \sum_k \frac{p(F_j^1 \cap F_k^n) - p(F_j^1)\ p(F_k^n)}{p(F_j^1)\ p(F_k^n)} p(F_k^n|i_x^n)\right) \quad \textbf{(S12)} \end{aligned}$$

If the contributions from each of the patches is sufficiently small, the higher order terms in (**S12**) can be ignored, so that

$$p(F_j^1|i_x) = p(F_j^1|i_x^1)\left(1 + \sum_k \sum_{n\neq 1}^{N} \frac{p(F_j^1 \cap F_k^n) - p(F_j^1)\ p(F_k^n)}{p(F_j^1)\ p(F_k^n)} p(F_k^n|i_x^n)\right) \qquad \textbf{(S13)}$$

Network interpretation

Using the notation:

$$W_{jk}^{1n} = \frac{p(F_j^1 \cap F_k^n) - p(F_j^1)\ p(F_k^n)}{p(F_j^1)\ p(F_k^n)} \qquad \textbf{(S14)}$$

(**S12**) becomes:

$$p(F_j^1|i_x) = p(F_j^1|i_x^1)\prod_{n\neq 1}^{N}\left(1 + \sum_k W_{jk}^{1n}\ p(F_k^n|i_x^n)\right) \qquad \textbf{(S15)}$$

and (**S13**) becomes:

$$p(F_j^1|i_x) = p(F_j^1|i_x^1)\left(1 + \sum_k \sum_{n\neq 1}^{N} W_{jk}^{1n}\ p(F_k^n|i_x^n)\right) \qquad \textbf{(S16)}$$

It follows that the activity of the neurons representing feature F_j^1 in image x is

$$f_{j,x}^1 = g\left(p(F_j^1|i_x)\right) = g\left(\frac{1}{\mathcal{N}_x^1} c_{j,x}^1 \prod_{n\neq 1}^{N}\left(1 + \sum_k W_{jk}^{1n}\ g^{-1}\left(c_{k,x}^n\right)\right)\right) \qquad \textbf{(S17)}$$

where $\mathcal{N}_x^1$ represents a normalization coefficient for patch 1 in image x (the implementation of which will be described in the next section) and $c_{k,x}^n = g(p(F_k^n|i_x^n))$ represents the classical receptive field response of the neuron.

Using a simple neural code mapping $g(y) = y$ in which the firing rates represent linearly the probability, (**S17**) becomes

$$f_{j,x}^1 = \frac{1}{\mathcal{N}_x^1} c_{j,x}^1 \prod_{n\neq 1}^{N}\left(1 + \sum_k W_{jk}^{1n} c_{k,x}^n\right) \qquad \textbf{(S18)}$$

and (**S13**) becomes,

$$f_{j,x}^1 = \frac{1}{\mathcal{N}_x^1} c_{j,x}^1 \left(1 + \sum_k \sum_{n\neq 1}^{N} W_{jk}^{1n}\ c_{k,x}^n\right) \qquad \textbf{(S19)}$$

(**S18**) (equivalently (**S17**)) can be interpreted in terms of a network as follows. Let us suppose that the term W_{jk}^{1n} can be interpreted as a synaptic weight. Then (**S18**) implies that the firing rate can be obtained by summing the appropriately weighted contributions from k neurons in each patch n via lateral connections and multiplying the contributions from all N patches with the classical RF response $c_{j,x}^1$. We have thus shown how a network of neurons can directly implement Bayes rule to integrate information from the surround.

In general, the formula for the synaptic weight is

$$W_{jk}^{mn} = \frac{p(F_j^m \cap F_k^n)}{p(F_j^m)p(F_k^n)} - 1 \qquad \textbf{(S20)}$$

Please note that the organism cannot measure the probabilities themselves in (**S20**) directly. But they can be estimated from observations of the environment given our defined neuronal code. An estimate can be

$$W_{jk}^{mn} = \frac{\left\langle f_{j,x}^{m} f_{k,x}^{n} \right\rangle_x}{\left\langle f_{j,x}^{m} \right\rangle_x \left\langle f_{k,x}^{n} \right\rangle_x} - 1 \quad (\mathbf{S21})$$

when x spans a representative set of natural scenes. Such a set of weights can be realized using a Hebbian-like learning in an unsupervised manner. However, the firing rate $f_{m,x}^{j}$ is dependent on W_{jk}^{mn}, which can lead to problems of stability. Therefore we use the best estimate the organism can have about probabilities of feature presence (e.g. $p(F_{k1}^{n1})$ without a recursive relation on the weights. Thus we computed these weights using the classical receptive field responses of the cells so that,

$$W_{jk}^{mn} = \frac{\left\langle c_{j,x}^{m} c_{k,x}^{n} \right\rangle_x}{\left\langle c_{j,x}^{m} \right\rangle_x \left\langle c_{k,x}^{n} \right\rangle_x} - 1 \quad (\mathbf{S22})$$

Normalization

As described in Results, our model incorporates two types of normalization that arise from requiring that probabilities sum to one. The first corresponds to the requirement that the classical RF responses to an image patch satisfy,

$$\sum_k c_{k,x}^{n} = 1 \quad \forall n, x \quad (\mathbf{S23})$$

This normalization is carried out over a spatial region the size of the classical RF. It can be implemented in a neuronal network in which a set of neurons responsible for normalization i) have a divisive effect on the pyramidal neurons, ii) are patch specific (have a classical RF of similar size to the pyramidal neurons), iii) are untuned, iv) inhibit equally all the pyramidal neurons in their image patch and v) receive inputs equal to the average of the inputs of the pyramidal neurons in the patch. These properties match well with those of the pyramidal targeting inter-neurons (PTI) category [4] and correspond quite well to the parvalbumin-expressing inter-neurons [5].

The second normalization leads to the requirement that the firing rates of neurons obtained after incorporating the contributions from the lateral connections satisfy,

$$\sum_k f_{k,x}^{n} = 1 \quad \forall n, x \quad (\mathbf{S24})$$

We can condense the effect of all the lateral connections into one term by defining,

$$L_{j,x}^{m} = \prod_{n \neq 1}^{N} \left(1 + \sum_k W_{jk}^{mn} \; f_{k,x}^{n} \right) \quad (\mathbf{S25})$$

The equation for the firing rate then becomes

$$f_{k,x}^{n} = \frac{1}{\mathcal{N}_x^n} c_{k,x}^{n} \; L_{k,x}^{n} \quad (\mathbf{S26})$$

with

$$\mathcal{N}_x^n = \sum_k c_{k,x}^{n} L_{k,x}^{n}, \quad (\mathbf{S27})$$

ensuring that (**S24**) is satisfied. This normalization is carried out over a spatial region extending out to 4 times the classical RF size, encompassing the spatial region corresponding to the extra classical RF. This normalization arises in our network effectively as a consequence of network interactions between Pyr, PV and SOM interneurons.

Distributions of model synaptic weights

The computation of weights using (**S22**) produces both positive and negative weights, with an approximately balanced average. This balance is in addition to the local normalization implemented in (**S3**). This can be seen in Figure S1a which shows the distribution of all $(18 \times 18 \times 43 \times 43)$ synaptic weights predicted by our model in the 4-dimensional array $W(k_1, k_2, \Delta x, \Delta y)$. The synaptic weight distribution follows a heavy-tailed distribution with a bias towards excitatory connections, that has been reported experimentally in cortex [6] and proposed to have various computational implications [7–9].

Figure S1b also shows the distribution of synaptic weights at each spatial location on 7×7 gridpoints that are separated by the size of the classical receptive field. Again, it can be seen that the distributions are centered around 0 at most spatial locations, indicating a balance between excitatory and inhibitory weights in our model. This is also evident from the left and right panels in Figure S2, which show the average synaptic weight across all pairs of 18×18 filters in each spatial location on the full 43×43 and the reduced 7×7 spatial grid (same grid as in Figure S1b) respectively.

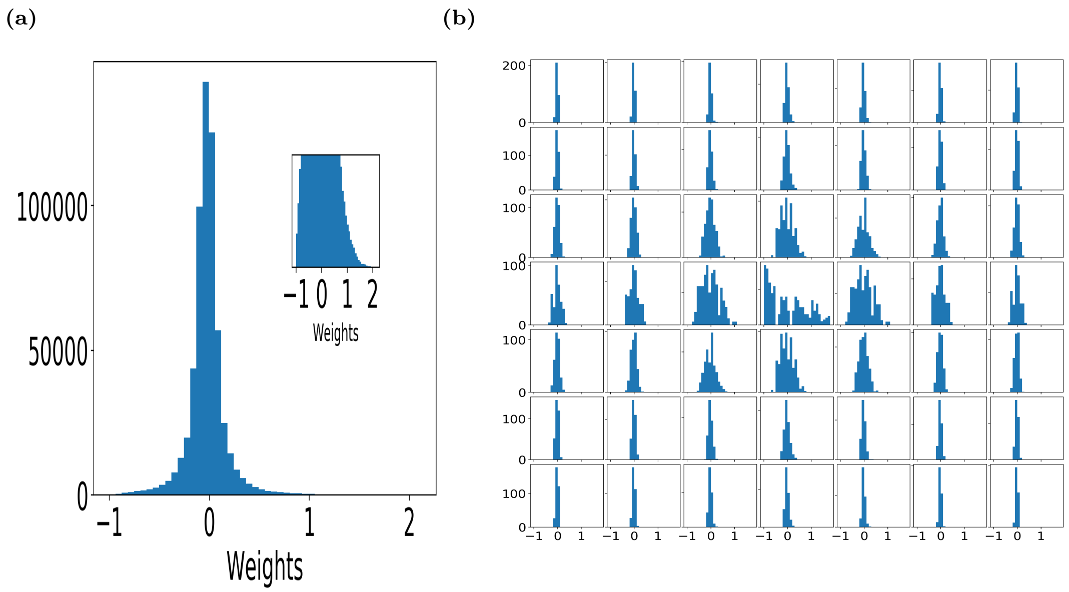

Figure S1: Distributions of synaptic weights. **Left:** Histogram of all computed synaptic weights for parameterized data filters showing a long-tailed distribution with a bias towards excitatory connections (mean $\mu = -0.02$ and standard deviation $\sigma = 0.19$). Inset shows a zoomed-in version to make the long-tailed nature more clear. **Right:** Histograms of synaptic weights at each spatial location on 7×7 gridpoints that are separated by the size of the classical receptive field. Note the vertical axes ranges are different across the rows.

Comparison with other models

Here we discuss some comparisons of our model with other normative and dynamical models relating contextual modulation of neuronal responses and lateral connectivity.

Dynamical models

A well-established dynamical model of lateral connectivity in V1 [10] uses lateral connections between neurons that are determined by the correlation between their classical receptive fields. This model allows us to define

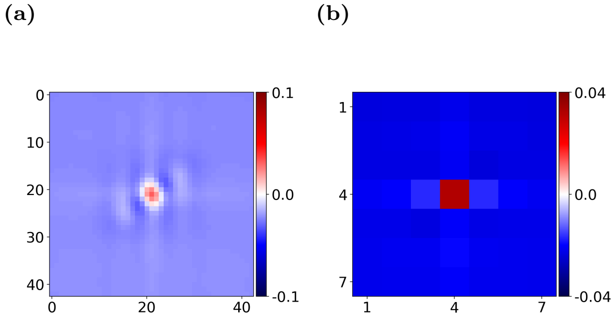

Figure S2: Spatial profile of the average of synaptic weights across all pairs of parameterized data filters showing balance between excitation and inhibition on average. **Left:** Mean on 43 x 43 grid. **Right:** Mean of synaptic weights at each spatial location on the same 7 × 7 grid as in Figure S1b.

synaptic weights between neurons i and j as,

$$\begin{aligned} W_{ij} = sign\left(c\left(i,j\right)\right)c\left(i,j\right)^{n_{pow}} \qquad n_{pow} \ \text{even} \\ W_{ij} = c\left(i,j\right)^{n_{pow}} \qquad n_{pow} \ \text{odd} \end{aligned} \tag{S28}$$

where $c(i,j)$ is the raw correlation between filters representing neurons i and j, $sign(x) = 1$ if x is positive and -1 if x is negative and n_{pow} is a parameter that determines connectivity strength as a function of correlation.

We used $n_{pow} = 6$ as in the original study [10] for direct comparison. Sample synaptic weights for the receptive field correlation model filters in all classes are shown in SI Figure (S3 top rows each panel) onto the first (leftmost) filter obtained using this procedure are shown in SI Figure (S3 third rows in each panel).

Results obtained from this model for $n_{pow} = 1$ are shown in SI Figure S3 bottom row of each panel. It can be seen that this model also produces like-to-like orientation and distance dependence of excitatory and inhibitory connections, qualitatively similar to our model. The panels in SI Figure S4 show the orientation and distance dependence of excitatory and inhibitory connections for the different classes of filters. We fit Gaussian functions (black dashed lines in SI Figure S4) to the distance dependence of synaptic weights from this model (see Methods). Please note that we were not able to find good Gaussian fits for the sharp filters with the correlation-based model, likely due to their very sharp fall-off with distance.

We also explored comparisons with the lateral connectivity profiles that have been established in dynamical network models of contour integration in primates [12, 13] (for brevity, we refer to these as the Li model [12] and the Piëch model [13]). Both these models provide explicit formulae for long range connectivity kernels between hypercolumns in primate V1 that facilitate smooth contours and suppress parallel flankers. Note that the excitatory and inhibitory connections in these models follow the rule of co-circularity and collinearity for the optimal connection strength at a given spatial location, in agreement with the statistics of natural images [14]. Using these expressions, we reproduced 4-D connectivity profiles for 8 orientations (from 0 to 315 degrees in steps of 45 degrees) on a 43 × 43 spatial grid, analogous to $W(k_1, k_2, \Delta x, \Delta y)$ generated with our model. Specifically, we used the same parameters and expressions as in [12] for the Li model, while the Piëch model [13] was parameterized using the expressions,

$$\cos_{fwhm}(\phi, \phi_{opt}, \phi_{fwhm}) = \begin{cases} \frac{1}{2}\cos\left(\pi\frac{\phi-\phi_{opt}}{\phi_{fwhm}}\right) + \frac{1}{2}, & |\phi - \phi_{opt}| < \phi_{fwhm} \\ 0, & |\phi - \phi_{opt}| \geq \phi_{fwhm} \end{cases} \tag{S29}$$

and,

$$L_{ee} = I_{ee} \exp\left(-\frac{\Delta x^2 + \Delta y^2}{2d_\sigma^2}\right) \cos_{fwhm}\left(-\theta_2, \theta_1, \frac{\pi}{4}\right) \cos_{fwhm}\left(\frac{|\theta_1| + |\theta_2|}{2}, 0, \frac{\pi}{4}\right)$$

$$L_{ie} = I_{ie} \exp\left(-\frac{\Delta x^2 + \Delta y^2}{2d_\sigma^2}\right) \cos_{fwhm}\left(\frac{\pi}{2} - \theta_2, \frac{\pi}{2} - \theta_1, \frac{\pi}{4}\right) \cos_{fwhm}\left(\frac{|\theta_1| + |\theta_2|}{2}, \frac{\pi}{2}, \frac{\pi}{4}\right) \quad \textbf{(S30)}$$

L_{ee} and L_{ie} represent the long range excitatory and inhibitory weights respectively, I_{ee} and I_{ie} are overall scale factors for the weights and d_σ is a parameter representing a falloff distance for the weights. θ_1 and θ_2 are angles between the preferred orientations of the two neurons and the line connecting them. If $\theta_{1,2}$ was larger than the angle of the connecting line, we reset $\theta_{1,2} \to \pi - \theta_{1,2}$. For the Piëch model, we used $d_\sigma = 10,\ I_{ee} = 1,\ I_{ie} = 1$.

SI Figure S5 shows the spatial profile for synaptic weights between a pair of neurons coding for a horizontal feature (represented by the filter in leftmost panel) for our model (second panel), the Li model (third panel) and the Piëch model (last panel). All three models produce qualitatively similar spatial profiles of excitatory and inhibitory weights for neurons preferring horizontal features. Additionally, SI Figure S6 shows that these models also qualitatively capture the like-to-like connectivity and distance dependence, similar to our model.

Normative models

Our model bears close resemblance to the MGSM (mixture of Gaussian scale mixtures) model of natural images proposed by Coen-Cagli, Dayan and Schwartz [15], which infers contextual interactions between the RF and surround that would lead to optimal coding of images. Analogous to our model, we mapped the effective interactions learned by their model onto a circuit and found that their model also qualitatively reproduces the like-to-like connectivity and distance dependence of positive and negative weights.

We used the software [16] made publicly available by the authors at http://dx.doi.org/10.6080/K0JM27JZ and obtained covariance matrices for RF interactions at four different relative spatial positions ($6,\ 12,\ 18\ \textit{and}\ 24$ pixels) of center and surround RFs. Other details were kept exactly the same including the number and types of center and surround filters. We used only the covariance matrices for interactions between center and surround RFs from their model for further analysis. Equating these with synaptic weights, we obtained a circuit mapping by splitting them into positive (excitatory) and negative (inhibitory) weights as in our model. Further analyses for the orientation and distance dependence of synaptic weights was carried out in an exactly identical manner as for our model.

SI Figure S7 shows (in a matrix layout) the spatial profile of synaptic weights learned by the model between even-phase vertical (0 deg) surround filters and even-phase center filters at four orientations corresponding to (0, 45, 90, 135) deg for the four relative spatial positions. SI Figure S8 shows the qualitatively similar like-to-like connectivity and distance dependence of both excitatory and inhibitory weights. Given our mapping into excitatory and inhibitory weights, it can be seen that this model also provides a rich profile of synaptic weights as a function of relative spatial locations and orientation preferences for pairs of neurons. Since exact quantities depend on details of the filters used (among others), we did not pursue a more in-depth comparison here.

Adding lateral connections to deep convolutional networks

MNIST image dataset

We trained and evaluated our models on the MNIST [18] dataset. MNIST contains grayscale images (28x28 pixels) of handwritten digits (10 classes, for the digits 0-9). MNIST contains a total of 70K images, split into a training set (60K images) and a test set (10K images). We used 10% of the training data (6K images) for validation.

To test the generalization of our models under noise perturbations, we added two types of noise to the original images: additive white Gaussian noise (AWGN) and salt-and-pepper noise (SPN). The mean of the AWGN was set to zero and the standard deviation varied in increasing levels of $\{0.1, 0.2, 0.3, 0.4, 0.5\}$. For

Model	Network architecture								
CNN	conv5-13		maxpool	conv5-26		maxpool	FC-50	FC-10	soft-max
CNNEx	conv5-10	*conv7-10*	maxpool	conv5-20	*conv3-20*	maxpool	FC-50	FC-10	soft-max

Table 1: Model architectures used for the experiments. CNN is the baseline model without lateral connections, and CNNEx is the model with lateral connections. The number of parameters for CNN and CNNEx were approximately matched to ensure fair comparison of the two models. Convolutional layers are denoted as "conv<receptive field size>-<number of channels>". Convolutional layers in italics represent recurrent lateral connections learned in an unsupervised manner. "maxpool" denotes max pooling using a 2x2 window and a stride of 2. "FC" denotes fully connected layers with the given number of units. The ReLU activation function is not shown for brevity.

the SPN, the fraction of noisy pixels varied in increasing levels of $\{0.1, 0.2, 0.3, 0.4, 0.5\}$. The addition of noise can be viewed as a random, non-targeted adversarial attack, which changes the input image in such a way that it will be classified incorrectly. The degree of misclassification is dependent on the noise level. Example stimuli from each dataset (original and noisy images) are shown in Figure 6 in the Main text.

Network architecture and training

We used a simple network architecture to study the influence of lateral connections in convolutional neuronal networks. The network consisted of two convolutional (conv) layers with the ReLU nonlinearity, each followed by a max-pooling (maxpool) layer with a 2x2 pooling window, which effectively downsamples the input by a factor of 2. Following the two convolutional layers are two fully-connected (FC) layers, with the final output passed through a soft-max nonlinearity for the ten classes in each dataset. The model architecture is shown in Table 1.

To train our models, we used stochastic gradient descent with a learning rate of 0.01 and a momentum value of 0.5. We used a minibatch size of 64, and trained our models for a total of ten epochs. We trained ten different instantiations of each model using different random seeds to ensure the robustness of our results. All experiments were performed using Pytorch (0.3.1) on a NVIDIA GTX 1080 Ti GPU.

Adding lateral connections

After the initial phase of supervised learning, we freeze the feedforward synaptic weights of the network. The classical receptive field response of a neuron representing feature j in layer l in patch m, given image x can be represented by the activation of a standard artificial neuron model:

$$c_{j,x}^{m,l} = \phi\left(b + \sum_k \sum_n U_{jk}^{mn} c_{k,x}^{n,l-1}\right) \qquad \textbf{(S31)}$$

where ϕ represents a nonlinear activation function, b represents a bias term, k represents features, n represents locations, and U_{jk}^{mn} are the feedforward synaptic weights from layer $l-1$ to layer l.

We then apply lateral connections within the first two convolutional layers of the network. Here, we drop the l superscript, since the proposed lateral connections are intracortical and occur within the same layer. The lateral connections are between neurons with the same feature (within-channel) and neurons with different features (between-channel). The activity of a neuron representing feature j in patch m, given image x can then be written as:

$$f_{j,x}^{m} = c_{j,x}^{m}\Big(1 + \alpha \sum_k \sum_{n \neq m} W_{jk}^{mn} c_{k,x}^{n}\Big) \qquad \textbf{(S32)}$$

where $f_{j,x}^{m}$ represents the full response of the neuron with contributions from extra-classical receptive fields, $c_{j,x}^{m}$ represents the classical receptive field response of the neuron, α represents a hyperparameter that tunes

the strength of the lateral connections, and W_{jk}^{mn} are the synaptic weights from surrounding neurons. The lateral connections have a modulatory effect on the feedforward response, and setting $\alpha = 0$ is equivalent to the base model with no lateral connections.

The synaptic weights are learned in an unsupervised manner using the following rule, derived in (**S22**) above:

$$W_{jk}^{mn} = \frac{\left\langle c_j^m c_k^n \right\rangle_x}{\left\langle c_j^m \right\rangle_x \left\langle c_k^n \right\rangle_x} - 1 \quad \textbf{(S33)}$$

where W_{jk}^{mn} is the synaptic weight between each pair (j, k) of features located at (m, n) and x spans a set of images. We used the same set of training images originally shown to the network during supervised training to learn the lateral connections. It is important to note that this formula differs from a Hebbian learning rule, in that only the covariance between the feedforward responses of neurons leads to changes in the lateral connections.

Validation and testing

Lateral connections had a spatial extent of 7x7 pixels in the first convolutional layer and 3x3 pixels in the second convolutional layer. We do not include any self-connections, so these were all set to zero. We chose the lateral connection hyperparameters α for each of the two convolutional layers based on a coarse grid search over the parameter range $\{0.1, 0.01, 0.001, 0.0001\}$ using the validation dataset.

We did not use lateral connections for the two fully-connected layers. We report final accuracies of each model on the original dataset and for all levels of the two different types of noise perturbations. All final results are averages over each of the 10 pre-trained models with different random seeds.

Phenomenology

We have shown that our model captures the distance-dependence and like-to-like nature of excitatory connectivity reported experimentally.

We now demonstrate two simple instances of contextual modulation (specifically, surround suppression) observed in experiments that we can reproduce using the learned lateral connections in our model.

The simplest form of surround suppression is perhaps the phenomenon of end-stopping. This refers to the reduction in firing rate of a neuron responding to an optimally oriented bar stimulus when the bar is extended beyond the classical RF boundary. Figure S11a shows an example neuron in our model exhibiting the same characteristic suppression with increasing bar length. Model neurons also show another form of surround suppression in which their response to a square-wave grating patch decreases with increasing patch size [19, 20]. Furthermore, the optimal patch size evoking the maximum response is larger at lower contrast. This is shown for an example model neuron in Figure S11b.

Both of these phenomena arise in the model as consequence of network interactions giving rise to a combination of subtractive and divisive inhibition [21]. Recall that the firing rate of a neuron in the network is related to the probability of a feature being present in the image at a given location ((**S1**)) and is calculated using (**S19**). We already normalized the classical RF responses ((**S1**)). To ensure that sum of all probabilities is still one, we implement (with $g(y) = y$) an additional normalization

$$\sum_k f_{k,x}^n = \sum_k p(F_k^n | i_x) = 1 \quad \forall n, x \quad \textbf{(S34)}$$

This is achieved by divisive normalization of activities over a spatial region extending out to 4 times the classical receptive field size, which encompasses the spatial region corresponding to the extra-classical RF.

An intuitive justification for this additional normalization can be provided as follows. In our model, network neurons integrate information about feature presence from surround neurons via lateral connections. Upon receiving information from the surround neurons, each neuron updates its estimate about the probability

of a feature being present in the image. The inclusion of this information and the subsequent normalization leads to a reshaping of neuronal responses that manifests experimentally in the form of extra-classical RF effects.

Divisive normalization of this nature has been proposed as a possible explanation for surround suppression [22]. Other models have used variants of divisive normalization such as a weighted (as opposed to uniform) divisive sum [23] or input-targeted divisive inhibition [24, 25] to explain extra-classical effects including surround SI suppression. SOM neurons have been implicated in mediating surround suppression in mouse visual cortex [19] as mentioned earlier. However, in our model, it is difficult to ascribe SOM neurons as being solely responsible for surround suppression arising from this divisive normalization. Instead, it arises in our model effectively as a consequence of network interactions between Pyr, PV and SOM interneurons [21].

Image Reconstruction

To explore if lateral connections might facilitate decoding of information from neuronal activity by downstream neuronal populations, we reconstructed natural images after adding Gaussian noise (to simulate neuronal noise) to the activities of the neurons (Figure S12b, S12c).

We constructed maps of activities for each filter, and used the inverted filters to reconstruct the original image from all neuron activities. For a given input image x, we calculated the effective activity $f_{k1,x}^{n1}$ of neuron coding for feature F_{k_1} at location n_1 using Eq. (**S1**). We convolved the activities computed using this equation with the inverses of the filters in our basis set. Specifically, the activity $f_{k1,x}^{n1}$ was convolved with the inverse of the filter coding for feature F_{k_1} (obtained by flipping this filter about the horizontal and vertical axes). The convolutions with all inverted filters were then summed together to obtain the reconstructed image. We only use synaptic weights from relative spatial locations that are separated by the size of the classical RF, in accordance with the assumption that surround patches provide information which is independent of the patch in the classical RF.

To quantify reconstruction fidelity, we calculated the Pearson correlation coefficient r between the input and reconstructed natural images. We find that the correlation coefficient is larger for reconstruction with all lateral connections than with feedforward connections only (distribution of differences in correlations shown in Figure S12d, mean $\mu = 6.35 \times 10^{-4}$, standard error of mean $sem = 5.95 \times 10^{-5}$, ttest with p-value $p = 3.86 \times 10^{-21}$). Figure S12 shows an example input image (Figure S12a) and the reconstructed images based on the activities from the classical RF alone (Figure S12b, $r = 0.597$) and including all lateral connections (Figure S12c, $r = 0.598$) respectively. Shuffling the entries in the connectivity matrix no longer see a significant difference between the distributions of the correlation coefficients with and without lateral connections (SI Figure S9, mean $\mu = 3.02 \times 10^{-5}$, standard error of mean $sem = 2.58 \times 10^{-5}$, ttest with p-value $p = 0.24$). This shows that the specific pattern of lateral connections (and not just their presence) is important. Although the mean of the difference between the distributions of the two correlation coefficients is low, we note that the model does not require any supervised training, and that lateral connections are present for only one set of features in one layer.

References

[1] H. Barlow. "Pattern recognition and the responses of sensory neurons". *Annals of the New York Academy of Sciences* 156 (2) (1969): pp. 872–881.

[2] T. J. Anastasio, P. E. Patton, and K. Belkacem-Boussaid. "Using Bayes' rule to model multisensory enhancement in the superior colliculus". *Neural Computation* 12 (5) (2000): pp. 1165–1187.

[3] R. P. Rao. "Bayesian computation in recurrent neural circuits". *Neural computation* 16 (1) (2004): pp. 1–38.

[4] X. Jiang et al. "Principles of connectivity among morphologically defined cell types in adult neocortex". *Science* 350 (6264) (Nov. 2015): aac9462–aac9462.

[5] C. K. Pfeffer et al. “Inhibition of inhibition in visual cortex: the logic of connections between molecularly distinct interneurons.” *Nature neuroscience* 16 (8) (Aug. 2013): pp. 1068–76.

[6] S. Song et al. “Highly nonrandom features of synaptic connectivity in local cortical circuits”. *PLoS biology* 3 (3) (2005): e68.

[7] J.-n. Teramae and T. Fukai. “Computational implications of lognormally distributed synaptic weights”. *Proceedings of the IEEE* 102 (4) (2014): pp. 500–512.

[8] G. Buzsáki and K. Mizuseki. “The log-dynamic brain: how skewed distributions affect network operations”. *Nature Reviews Neuroscience* 15 (4) (2014): p. 264.

[9] R. Iyer et al. “The influence of synaptic weight distribution on neuronal population dynamics”. *PLoS computational biology* 9 (10) (2013): e1003248.

[10] T. W. Troyer et al. “Contrast-invariant orientation tuning in cat visual cortex: thalamocortical input tuning and correlation-based intracortical connectivity”. *Journal of Neuroscience* 18 (15) (1998): pp. 5908–5927.

[11] S. Durand et al. “A comparison of visual response properties in the lateral geniculate nucleus and primary visual cortex of awake and anesthetized mice”. *Journal of Neuroscience* 36 (48) (2016).

[12] Z. Li. “A neural model of contour integration in the primary visual cortex.” *Neural computation* 10 (4) (May 1998): pp. 903–40.

[13] V. Piëch et al. “Network model of top-down influences on local gain and contextual interactions in visual cortex”. *Proceedings of the National Academy of Sciences* 110 (43) (2013): E4108–E4117.

[14] M. Sigman et al. “On a common circle: natural scenes and Gestalt rules”. *Proceedings of the National Academy of Sciences* 98 (4) (2001): pp. 1935–1940.

[15] R. Coen-Cagli, P. Dayan, and O. Schwartz. “Cortical surround interactions and perceptual salience via natural scene statistics”. *PLoS computational biology* 8 (3) (2012): e1002405.

[16] R. Coen-Cagli, P. Dayan, and O. Schwartz. “MatLab tools for building Mixture of Gaussian Scale Mixture (MGSM) models, and perform inference and learning”. *CRCNS.org* (2016).

[17] R. Coen-Cagli, P. Dayan, and O. Schwartz. “Cortical Surround Interactions and Perceptual Salience via Natural Scene Statistics.” *PLoS computational biology* 8 (3) (Mar. 2012): e1002405.

[18] Y. LeCun. “The MNIST database of handwritten digits”. *http://yann. lecun. com/exdb/mnist/* ().

[19] H. Adesnik et al. “A neural circuit for spatial summation in visual cortex.” *Nature* 490 (7419) (Oct. 2012): pp. 226–31.

[20] M. W. Self et al. “Orientation-tuned surround suppression in mouse visual cortex”. *Journal of Neuroscience* 34 (28) (2014): pp. 9290–9304.

[21] B. A. Seybold et al. “Inhibitory actions unified by network integration”. *Neuron* 87 (6) (2015): pp. 1181–1192.

[22] M. Carandini and D. J. Heeger. “Normalization as a canonical neural computation”. *Nature Reviews Neuroscience* 13 (1) (2012): p. 51.

[23] O. Schwartz and E. P. Simoncelli. “Natural signal statistics and sensory gain control”. *Nature neuroscience* 4 (8) (2001): p. 819.

[24] T. Lochmann, U. A. Ernst, and S. Deneve. “Perceptual inference predicts contextual modulations of sensory responses”. *Journal of neuroscience* 32 (12) (2012): pp. 4179–4195.

[25] M. Chalk et al. “Sensory noise predicts divisive reshaping of receptive fields”. *PLoS computational biology* 13 (6) (2017): e1005582.

(a) Data filters

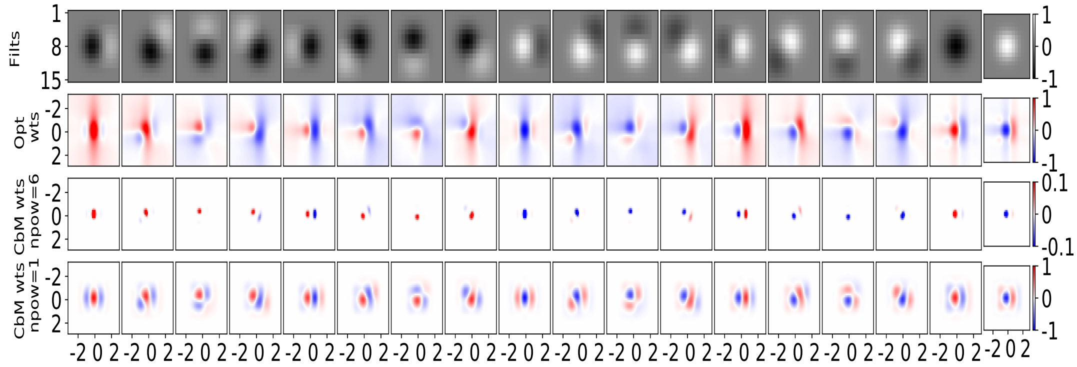

(b) Gabor filters

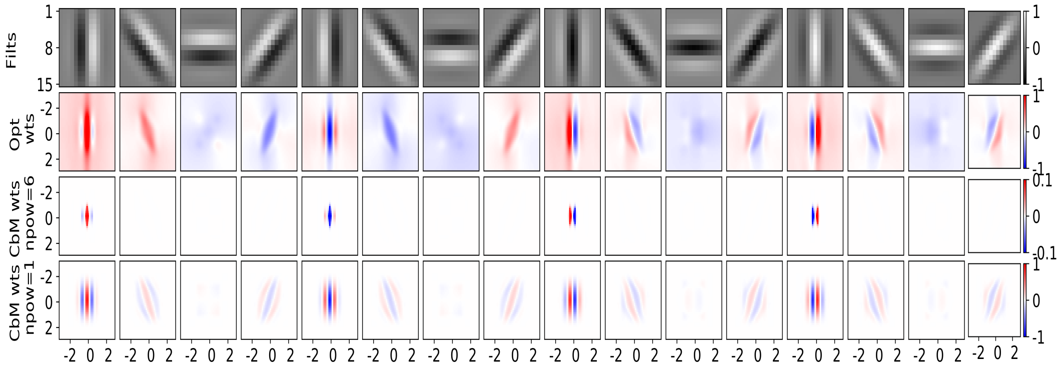

(c) Sharp/banded filters

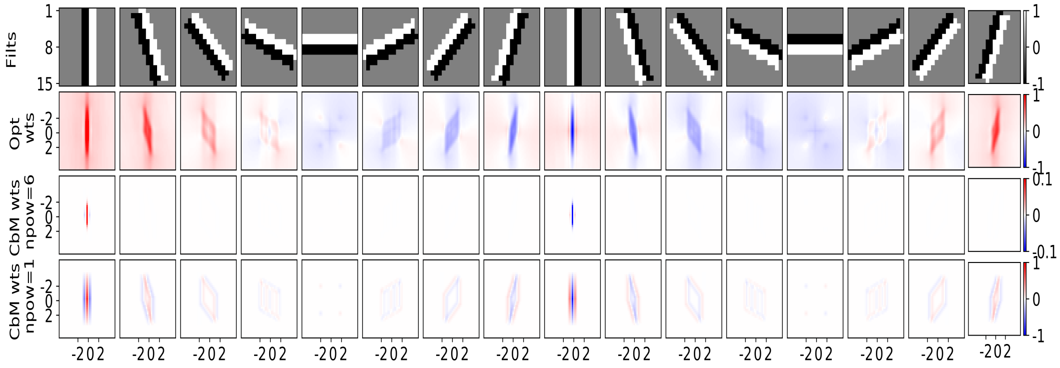

Figure S3: Comparison of synaptic weights from our model (Opt wts) and the receptive field correlation model [10] with parameter $n_{pow} = 6$ (CbM wts npow = 6) and $n_{pow} = 1$ (CbM wts npow = 1) for all **(a)**18 parameterized data filters, **(b)**16 Gabor filters and **(c)**16 sharp/parameterized filters. For each filter class, the four rows represent: **Top:** All basis filters, generated on a 15 × 15 grid. **Second:** Synaptic weights onto the target neuron representing the left-most filter in above located at position $\vec{x}_1$ from the neurons representing filters $k_2 = (1, ..., 18)$ at position $\vec{x}_2$. Synaptic weights were calculated in each direction around k_1 using (**S22**). **Third:** Synaptic weights as in the second row, calculated using the correlation between classical receptive fields of each pair of neurons with $n_{pow} = 6$ (see Methods). **Bottom:** Synaptic weights calculated using the correlation between classical receptive fields of each pair of neurons with $n_{pow} = 1$ (see Methods). For the last 3 rows, the axes represent distances from the center in terms of the RF size. Note that the colorbar for the third row ranges from -0.1 to 0.1.

(a) Data filters **(b)** Gabor filters **(c)** Sharp/banded filters

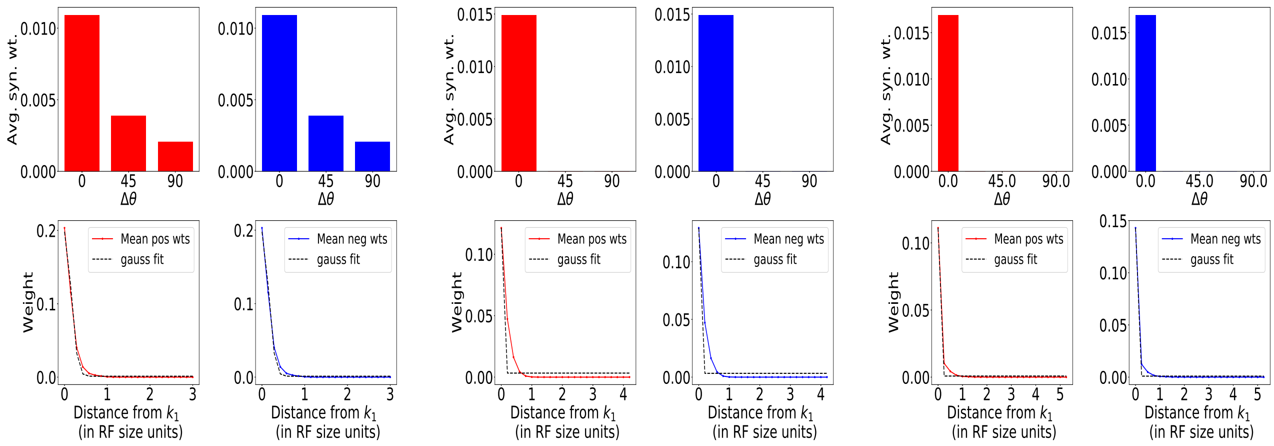

Figure S4: Orientation and distance dependence of connections from the correlation-based model [10] with parameter $n_{pow} = 6$ for **a)** parameterized data filters from mouse V1 [11], **b)** Gabor filters and **c)** Sharp/banded filters. Sharp filters were generated with a spacing $\delta\theta = 22.5$ deg as opposed to 45 deg for the data and Gabor filters. For each filter class in **a,b,c) Left top:** Predicted average positive synaptic weights from the correlation-based model as a function of difference in orientation. **Left bottom:** Dependence of mean positive synaptic weights (points) on distance from RF center of target filter k_1 and corresponding Gaussian fits for the positive weights (dashed black lines). **Right column:** Same as in left column, for negative synaptic weights. In all plots, red bars/lines represent positive weights and blue bars/lines represent negative weights.

(a) Example filter **(b)** Our model **(c)** Li model **(d)** Piëch model

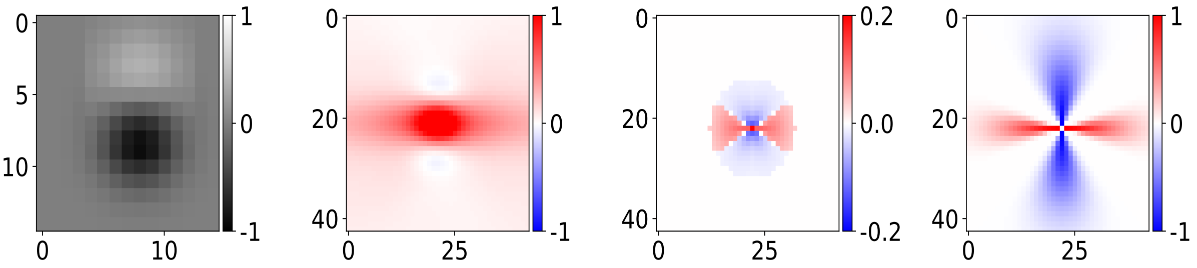

Figure S5: Our model shows qualitatively similar spatial profile of synaptic weights as in the dynamical models developed to explain contour integration in primates. **(a)** Example parameterized data filter representing a neuron coding for horizontal features from our filter set, **(b)** Weights predicted by our model between pairs of neurons represented by the filter in panel (a), **(c)** Corresponding weights between pairs of neurons coding for horizontal features from the dynamical model of Li [12] and **(d)** analogous weights from the dynamical model of Piëch et. al [13].

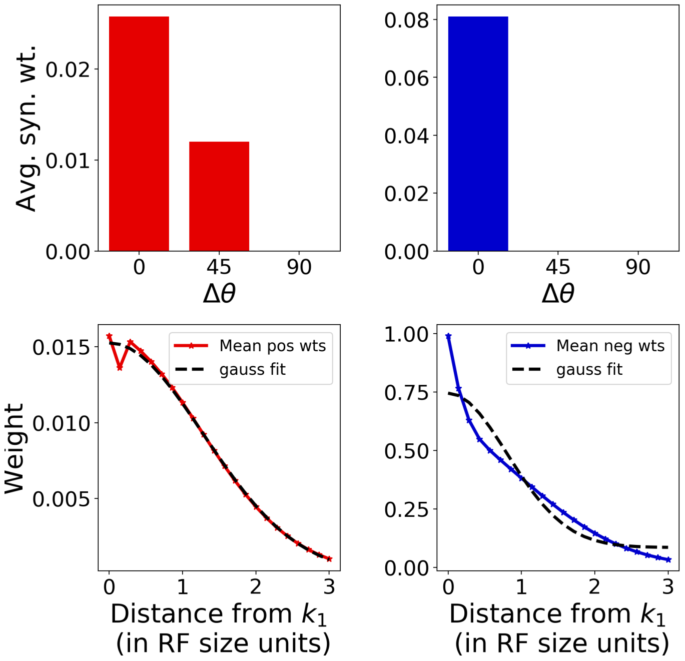

Figure S6: Orientation and distance dependence of excitatory (red bars/lines) and inhibitory (blue bars/lines) connections from the Piëch dynamical model [13].

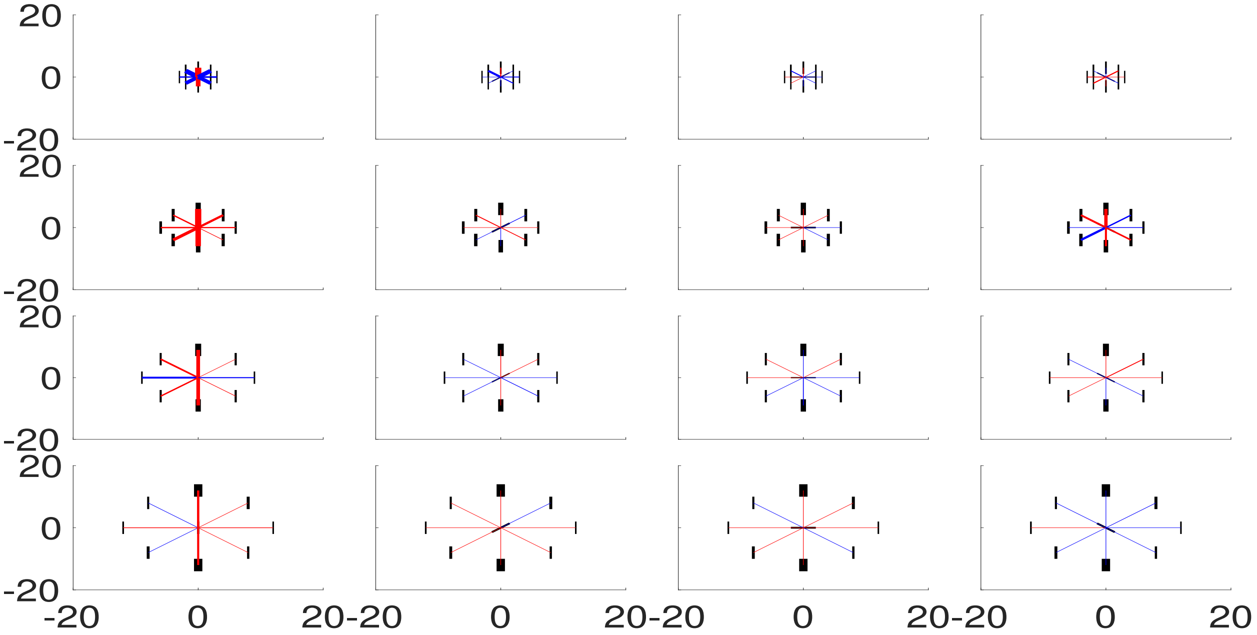

Figure S7: Spatial profile of covariances (which we identify with synaptic weights) between even-phase center and surround filters for vertical surround filters learned by the MGSM model [17]. We use the same representation scheme as in the original study. Black bars denote the orientation and relative position of the RFs; bar thickness is proportional to the variance. The thickness of the red (blue) lines connecting the center and surround bars is proportional to the positive (negative) covariance respectively (normalized by the maximum variance among the 4 distances). Rows correspond to different relative distances between center and surround filters ((6, 12, 18, 24 pixels) respectively from top to bottom), while columns correspond to different orientations of the center filters ((0, 45, 90, 135 deg) respectively from left to right). The leftmost column corresponds to their configuration ξ_0.

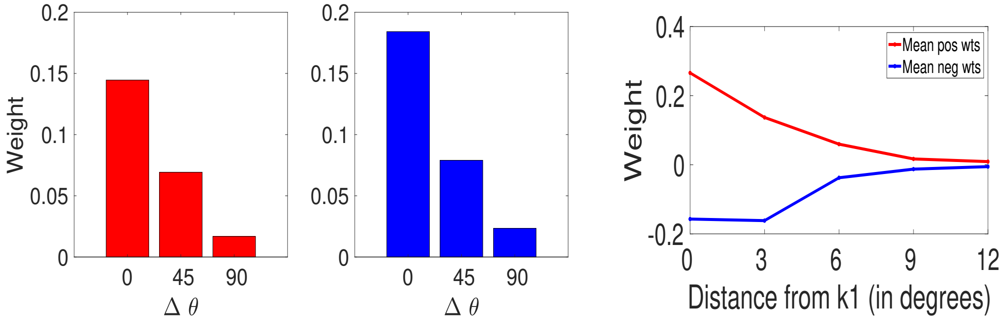

Figure S8: Orientation and distance dependence of covariances/weights from the MGSM model for even phase center and surround filters [15]. **Left and middle panels:** Predicted average positive and negative synaptic weights respectively, as as function of difference in orientation tuning. **Rightmost panel** Mean covariances (synaptic weights) as a function of distance from center filter. Similar results hold for odd phase filters (not shown here).

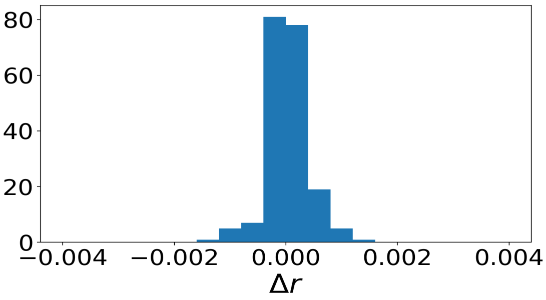

Figure S9: Distribution of difference in Pearson correlation coefficients computed with shuffled lateral connections and only feedforward connections for the same test set of 200 natural images as in the main text (mean $\mu = 3.02 \times 10^{-5}$, standard error of mean $sem = 2.58 \times 10^{-5}$, ttest with p-value $p = 0.24$)

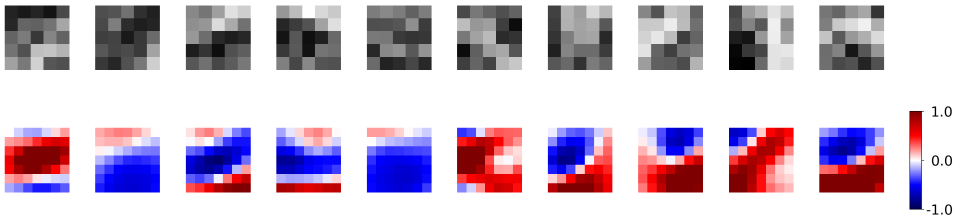

Figure S10: Example lateral connections learned on the MNIST dataset. The first row shows filters for the first convolutional layer learned in a supervised manner. The second row shows lateral connections from each filter onto the first filter in each row learned in an unsupervised manner. The learned filters were 5x5 and the optimal lateral connections were 7x7.

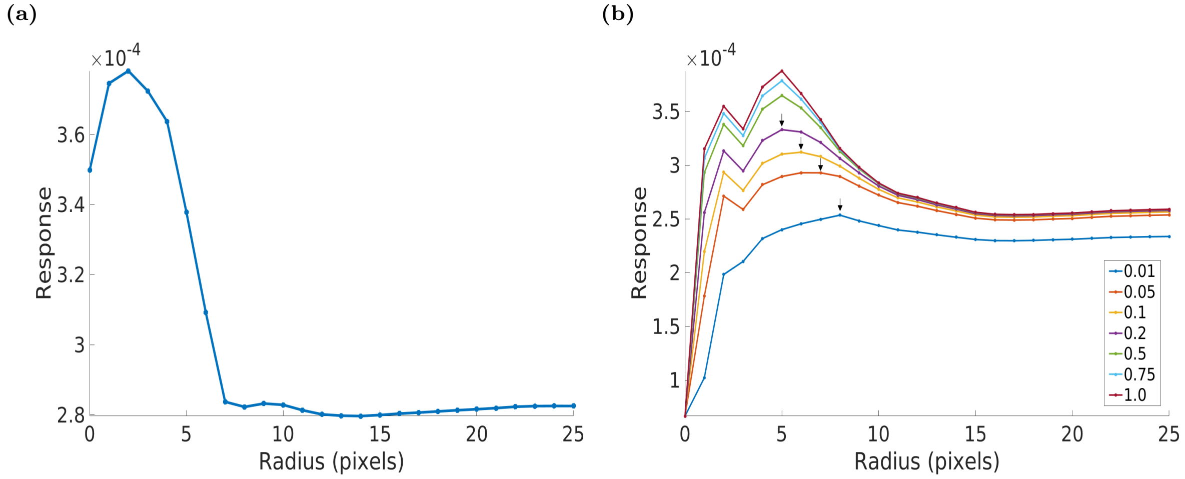

Figure S11: Model neurons exhibit surround suppression and RF expansion. **a)** Response of a neuron in our network model to a bar of increasing length that is consistent with end stopping behavior observed in experiments. **b)** Response of a model neuron as a function of stimulus size for a grating stimulus with different contrasts (represented by the different colored lines). In accordance with physiology, the neuron shows suppression with increasing stimulus size and increase in optimal stimulus size for lower contrasts. The small black arrows represent the maxima of the tuning curves at the different contrasts, with the four highest contrasts all having their maxima at the same stimulus size.

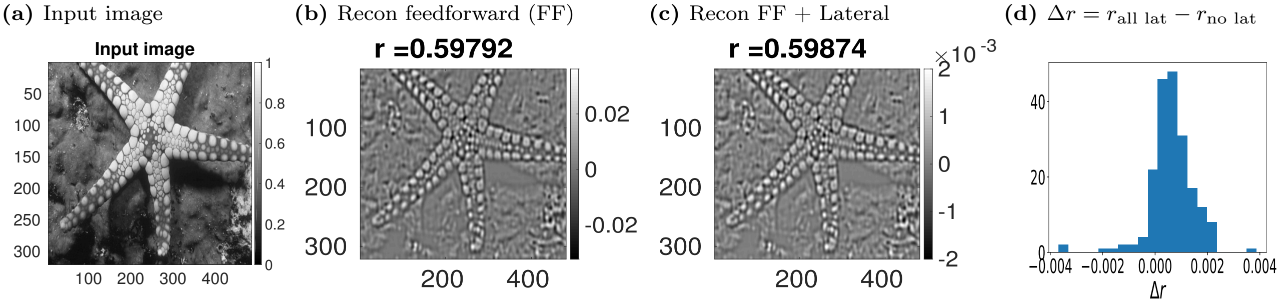

Figure S12: Image reconstruction from noisy representations. Inclusion of lateral connections leads to higher reconstruction/decoding fidelity for natural images. **(a)** Input natural image. **(b)** Input image reconstructed from noisy feedforward activities alone. **(c)** Input image reconstructed from combining noisy feedforward activities with contributions from lateral connections. The value of the Pearson correlation coefficient between input and reconstructed image is specified on top of the respective figures. **(d)** Distribution of difference in correlation coefficients computed with all lateral connections and without lateral connections for a *test* set of 200 natural images from the BSDS dataset (mean $\mu = 6.35 \times 10^{-4}$, standard error of mean $sem = 5.95 \times 10^{-5}$, ttest with p-value $p = 3.86 \times 10^{-21}$)
